# Metabolic-epigenetic reprogramming *via* the PLOD1–PFKP axis drives cisplatin resistance in HNSCC

**DOI:** 10.1016/j.apsb.2026.03.053

**Published:** 2026-04-03

**Authors:** Xinyuan Zhao, Xin Liao, Yunfan Lin, Xu Chen, Pei Lin, Ye Lu, Jiarong Zheng, Meiyan Zou, Bing Guo, Li Cui

**Affiliations:** a Stomatological Hospital, School of Stomatology, Southern Medical University, Guangzhou 510280, China; b Department of Dentistry, The First Affiliated Hospital, Sun Yat-sen University, Guangzhou 510080, China; c School of Dentistry, University of California, Los Angeles, CA 90095, USA

**Keywords:** Cisplatin resistance, Epigenetic regulation, Glycolysis, Head and neck squamous cell carcinoma, Metabolic reprogramming, PLOD1, PFKP, TGFBR2

## Abstract

Cisplatin remains a cornerstone of treatment for head and neck squamous cell carcinoma (HNSCC), yet its therapeutic efficacy is often undermined by acquired resistance. Here, we identify the PLOD1–PFKP–glycolysis axis as a central driver of cisplatin resistance. PLOD1 is significantly upregulated in cisplatin-resistant tumors and correlates with poor prognosis. Mechanistically, PLOD1 stabilizes the glycolytic enzyme PFKP by promoting its AKT-mediated phosphorylation at serine 386 *via* the HSP90–AKT complex, thereby enhancing glycolytic flux. This metabolic reprogramming facilitates stem-like properties and sustains epithelial–mesenchymal transition (EMT). Furthermore, increased intracellular acetyl-CoA levels driven by this axis promote histone H3K27 acetylation at the *TGFBR2* promoter, thereby activating TGF-*β* signaling. Genetic depletion or nanoparticle-mediated silencing of PLOD1 reverses EMT and stemness features, restores cisplatin sensitivity, and impairs tumor growth and metastasis *in vivo*. These findings reveal a PLOD1–PFKP–glycolysis axis as the principal driver of cisplatin resistance, which coordinates metabolic and epigenetic alterations to promote tumor plasticity. Targeting this axis offers a promising strategy to overcome chemoresistance in HNSCC.

## Graphical abstract

PLOD1, *via* the HSP90–AKT complex, stabilizes PFKP through AKT-mediated phosphorylation, enhancing glycolysis and acetyl-CoA production, activating H3K27ac-dependent TGFBR2 expression, and driving EMT, stemness, and cisplatin resistance in HNSCC.

**Figure:**
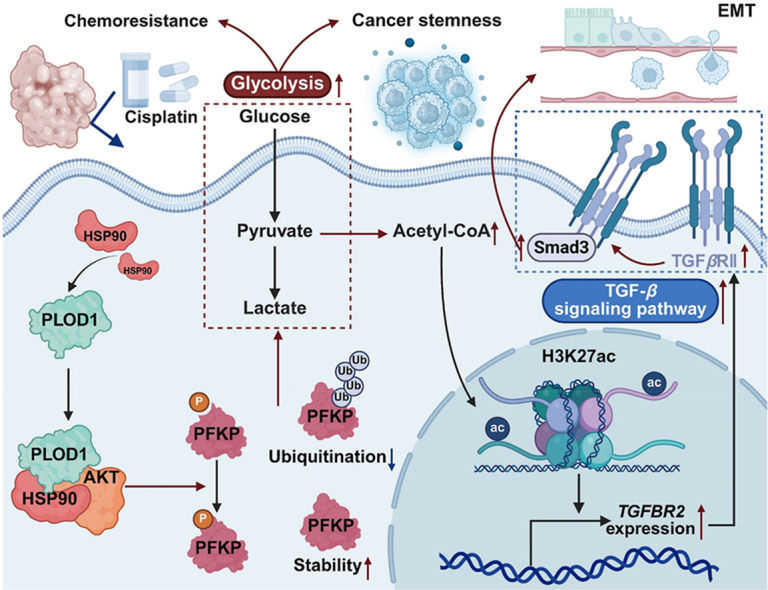

## 1 Introduction

Head and neck squamous cell carcinoma (HNSCC) represents the sixth most common malignancy worldwide and is associated with substantial morbidity and mortality^1^^,^^2^. Despite advances in surgical techniques, radiotherapy, and molecular-targeted therapies, platinum-based chemotherapy remains a cornerstone of treatment for patients with locally advanced or recurrent/metastatic HNSCC^3^^,^^4^. Among these agents, cisplatin is widely used as a first-line chemotherapeutic; however, its clinical efficacy is frequently hampered by the development of drug resistance, which contributes to treatment failure and poor prognosis^5^^,^^6^. Tumor relapse following cisplatin therapy is often characterized by enhanced epithelial–mesenchymal transition (EMT), enrichment of cancer stem-like cells, and metabolic reprogramming, all of which foster an aggressive and treatment-refractory phenotype^7^, ^8^, ^9^. Accordingly, elucidating the molecular mechanisms that confer cisplatin resistance and identifying novel therapeutic vulnerabilities are of urgent clinical importance for improving HNSCC patient outcomes.

Accumulating evidences indicate that metabolic reprogramming is a hallmark of cisplatin-resistant tumors^10^^,^^11^. Altered cellular metabolism not only sustains the bioenergetic and biosynthetic demands of proliferating cancer cells but also modulates redox homeostasis, epigenetic remodeling, and apoptotic signaling, thereby fostering chemoresistance^12^, ^13^, ^14^. In addition to glycolytic enzymes such as hexokinase 2, metabolic regulators involved in cholesterol synthesis (*e.g.*, SQLE) and folate-mediated one-carbon metabolism (*e.g.*, MTHFD1L) have also been implicated in drug resistance across various malignancies^6^^,^^15^. Nevertheless, how metabolic enzymes contribute to chemoresistance in HNSCC remains incompletely understood^16^^,^^17^. In particular, whether and how specific enzymes orchestrate cisplatin resistance through interconnected metabolic, epigenetic, and stemness-related circuits has yet to be fully elucidated. Uncovering these links could provide mechanistic insights and reveal actionable therapeutic vulnerabilities.

In this study, we identify procollagen-lysine, 2-oxoglutarate 5-dioxygenase 1 (PLOD1) as a key metabolic regulator that promotes cisplatin resistance in HNSCC. PLOD1 is markedly upregulated in cisplatin-resistant tumors and correlates with poor prognosis. Mechanistically, PLOD1 interacts with and protects PFKP from ubiquitin-mediated degradation by promoting its AKT-dependent phosphorylation at serine 386, a process facilitated through stabilization of the HSP90–AKT complex. This stabilization promotes glycolytic reprogramming and acetyl-CoA accumulation, which in turn supports cancer stem-like phenotypes and survival under cisplatin stress. Importantly, the metabolic–epigenetic coupling downstream of PFKP also enhances histone H3K27 acetylation at the *TGFBR2* promoter, contributing to EMT maintenance as an auxiliary pathway. Collectively, our data identify the PLOD1–PFKP–glycolysis axis as the principal driver of cisplatin resistance; by elevating acetyl-CoA and histone acetylation, this axis activates TGFBR2-centred EMT signaling, which in turn augments tumor plasticity.

## 2 Materials and methods

### 2.1 Patients and clinical samples

Archived tumor specimens and corresponding clinical data were collected from patients with HNSCC who underwent primary surgical resection. All diagnoses were histopathologically confirmed. Patients with a history of multiple primary malignancies or incomplete clinical or follow-up information were excluded. Tissue samples were either snap-frozen in liquid nitrogen and stored at −80 °C, or fixed in formalin and embedded in paraffin for subsequent analyses. For those who received induction chemotherapy prior to surgery, the treatment protocol consisted of intravenous docetaxel (75 mg/m^2^) and cisplatin (75 mg/m^2^) on Day 1, followed by a 120 h continuous infusion of 5-fluorouracil (750 mg/m^2^/day) from Day 1 to Day 5. Two cycles were administered at 3-week intervals. This study adhered to the principles of the Declaration of Helsinki and received approval from the Ethics Committee of the First Affiliated Hospital of Sun Yat-sen University (IRB Number: 2022-056).

### 2.2 Bioinformatic analysis of TCGA and GEO datasets

Genome-wide gene expression datasets comparing tumor tissues with adjacent non-tumor tissues, normal mucosa, or precancerous lesions were obtained from the Gene Expression Omnibus (GEO; https://www.ncbi.nlm.nih.gov/geo/). The GEO series used included: GSE23036, GSE25099, GSE27020, GSE30784, GSE30788, GSE31056, GSE34105, GSE37991, GSE40774, GSE41613, GSE51985, GSE55550, GSE58911, GSE65858, GSE6631, GSE85195, GSE117973, GSE127165, GSE136037, GSE142083, and GSE143224. RNA-seq data and corresponding clinical information for 33 cancer types were retrieved from The Cancer Genome Atlas (TCGA) *via* the Genomic Data Commons portal (https://gdc.cancer.gov/). To identify metabolic enzymes associated with HNSCC progression, transcriptomic data from the TCGA-HNSCC cohort were analyzed to screen for genes that were significantly upregulated in tumors and negatively correlated with patient survival.

Differential expression analysis of *PLOD1* between tumor and non-tumor tissues was performed using the TCGA-HNSCC and GEO datasets. The X-tile software (https://medicine.yale.edu/lab/rimm/research/software/) was employed to determine the optimal cutoff value for stratifying cancer patients into high and low *PLOD1* expression groups. For gene set enrichment analysis (GSEA), patients were grouped based on median *PLOD1* expression levels, and GSEA software was used to assess the statistical significance of predefined gene sets across expression subgroups.

### 2.3 Cell culture

The UMSCC-1 (SCC-1), UMSCC-23 (SCC-23), UMSCC-5 and UMSCC-6 cell lines were obtained from the University of Michigan. UM-1, UM-2 and NHOK cells were provided by the University of California, Los Angeles. CAL-27, NHEK and HEK293 cells were purchased from the American Type Culture Collection (ATCC). All cell lines were authenticated by short tandem repeat (STR) profiling and confirmed to be free of mycoplasma contamination. HNSCC cell lines were cultured in high-glucose DMEM supplemented with 10% fetal bovine serum and 100 U/mL penicillin–streptomycin, whereas HEK293 cells were maintained in MEM-*α* with the same supplements. NHOKs and NHEKs were grown in EpiLife medium containing human keratinocyte growth supplement (Invitrogen, Carlsbad, CA, USA). All cells were maintained at 37 °C in a humidified incubator with 5% CO_2_.

### 2.4 Plasmid, siRNA, lentivirus, and the transfection

Short hairpin RNAs (shRNAs) targeting PFKP were cloned into the LV3-pGLV-h1-GFP-puro lentiviral vector (GenePharma, Shanghai, China), and full-length PLOD1 cDNA was cloned into the pGCL-GFP vector (GeneChem, Shanghai, China) for overexpression studies. Double-stranded oligonucleotides encoding sgRNA sequences targeting PLOD1 were cloned into the BmsBI-digested LentiCRISPRv2 plasmid. Lentiviral particles were produced by co-transfecting HEK293T cells with expression and packaging plasmids, and after 72 h, viral supernatants were collected, filtered, and concentrated by ultracentrifugation. HNSCC cells were transduced with the lentivirus at appropriate MOIs, followed by puromycin selection for stable cell line establishment. The target sequences of shRNA and sgRNA were listed in Supporting Information Table S1. For plasmid transfections, C-terminal Flag-tagged full-length PLOD1 and its truncations (aa 1–90 to 631–727) were cloned into the KpnI/EcoRI sites of the pcDNA3.1(+) vector, while N-terminal HA-tagged full-length PFKP and its truncations (aa 1–399 to 400–784) were cloned into the BamHI/EcoRI sites of the same vector. Cancer cells were transfected with these plasmids using Lipofectamine 3000 (Invitrogen) according to the manufacturer's instructions. For siRNA transfection, siRNAs were transfected into HNSCC cells using RNAiMax (Invitrogen) according to the manufacturer's instructions.

### 2.5 Quantitative real-time PCR

Total RNA was extracted using the Quick-RNA™ extraction kit (Zymo Research, Irvine, CA, USA) following the manufacturer's instructions. cDNA was synthesized using SuperScript™ III reverse transcriptase (Invitrogen), and quantitative PCR was performed with SYBR Green I Master Mix (Roche, Indianapolis, IN, USA) on a LightCycler® 96 instrument (Roche). Relative gene expression was calculated using the 2^–ΔΔCt^ method, with *β*-actin as the internal control. Primer sequences are provided in Table S1.

### 2.6 Western blotting

Protein samples were resolved on 4%–20% SDS–polyacrylamide gradient gels and transferred onto 0.2 μm PVDF membranes using the Trans-Blot Turbo system (Bio-Rad, Hercules, CA, USA). Membranes were blocked for 10 min at room temperature (EpiZyme, Shanghai, China), incubated overnight at 4 °C with primary antibodies, and washed five times with TBST. HRP-conjugated secondary antibodies (Proteintech, Chicago, IL, USA) were applied for 1 h at room temperature. Signal detection was performed using the Amersham ECL Prime Western Blotting Detection Reagent (Cytiva, Marlborough, MA, USA), and band intensity was quantified using ImageJ software (NIH, Bethesda, MD, USA). The primary antibodies used in this study are as follows: PLOD1 (Proteintech), *β*-actin (Proteintech), Bcl-2 (Proteintech), Bax (Proteintech), ALDH1A1 (Proteintech), SOX2 (Proteintech), Nanog (Proteintech), CD133 (Proteintech), CD44 (Proteintech), Vimentin (Proteintech), E-cadherin (Proteintech), *N*-cadherin (Proteintech), *p*-Smad2 (Cell Signaling Technology, Danvers, MA, USA), Smad2 (Proteintech), *p*-Smad3 (Cell Signaling Technology), Smad3 (Proteintech), PFKP (Proteintech), GLUT1 (Proteintech), HIF-1*α* (Proteintech), HK2 (Thermo Fisher Scientific, Waltham, MA, USA), ENO1 (Abcam, Cambridge, UK), PKM2 (Cell Signaling Technology), LDHA (Cell Signaling Technology), TGF*β*RI (Santa Cruz Biotechnology, Santa Cruz, CA, USA), TGF*β*RII (Santa Cruz Biotechnology), TGF*β*RIII (Bioss, Beijing, China), HSP90 (Proteintech), Histone H3 (Proteintech), H3K27ac (Abcam), ATP1A1(Proteintech), and ACLY (Proteintech).

Cell-surface proteins were isolated using a modified Sulfo-NHS-SS-Biotin labeling approach. Briefly, cells were washed with ice-cold PBS and incubated with EZ-Link™ Sulfo-NHS-SS-Biotin (Thermo Fisher Scientific) at 4 °C to selectively label extracellular lysine residues. Unreacted biotin was quenched with glycine-containing buffer, and cells were lysed in a non-denaturing lysis buffer. Biotinylated proteins were captured using NeutrAvidin agarose, washed extensively, and eluted under reducing conditions before immunoblot analysis.

### 2.7 Colony formation assay

Cells with the indicated treatment were seeded in 6-well plates at a density of 3000 cells per well and cultured for 2 weeks. Colonies were fixed with 4% paraformaldehyde and stained with 0.5% crystal violet for visualization.

### 2.8 Drug sensitivity assay

Cells with the indicated genetic modifications were seeded into 96-well plates and exposed to a range of cisplatin concentrations. Cell viability was assessed by MTT assay. Briefly, 20 μL of MTT solution (5 mg/mL) was added to each well and incubated at 37 °C for 4 h. Formazan crystals were dissolved in 200 μL of DMSO, and absorbance was measured at 570 nm using a microplate reader. Viability was expressed relative to untreated controls, and IC_50_ values were determined by nonlinear regression analysis.

### 2.9 Matrigel invasion assay

Matrigel invasion assay was conducted using Transwell chambers with 8 μm pore membranes (Costar, Cambridge, MA, USA). Following serum starvation, cells were seeded into the upper chamber in serum-free medium, while the lower chamber was filled with complete medium as a chemoattractant. After 48 h of incubation, non-invading cells were removed from the upper membrane surface with a cotton swab. Invaded cells on the lower surface were fixed with 4% paraformaldehyde and stained with 0.5% crystal violet. Images were acquired from six random fields per chamber, and cell invasion was quantified by measuring the average invaded area using ImageJ software (NIH, Bethesda, MD, USA).

### 2.10 Annexin V/PI apoptosis assay

To assess apoptosis, treated cells were trypsinized, collected, and transferred to FACS tubes. After washing with ice-cold PBS, cells were resuspended in 100 μL of binding buffer and incubated with 5 μL annexin V-APC (Thermo Fisher Scientific) for 15 min at 25 °C in the dark. Samples were then washed, resuspended in binding buffer, and stained with 5 μL propidium iodide. Analysis was performed within 30 min using a DxFLEX flow cytometer (Beckman Coulter, Inc., Brea, CA, USA).

### 2.11 Sphere formation assay

Briefly, single-cell suspensions were seeded in ultralow-attachment plates (Corning). Cells were cultured in serum-free DMEM/F12 medium (Gibco, Grand Island, NY, USA) supplemented with 1% B27 (Invitrogen), 1% N_2_ (Invitrogen), 20 ng/mL epidermal growth factor (EGF) (PeproTech, Rocky Hill, NJ, USA), and 10 ng/mL basic fibroblast growth factor (bFGF) (PeproTech). Cultures were maintained at 37 °C in a humidified atmosphere with 5% CO_2_ until visible spheres were formed. Sphere formation was quantified based on total sphere area using ImageJ software.

### 2.12 ALDEFLUOR™ assay

ALDH activity was assessed using the ALDEFLUOR™ assay kit (StemCell Technologies, Vancouver, BC, Canada) according to the manufacturer's instructions. For each sample, a parallel aliquot was treated with the ALDH-specific inhibitor diethylaminobenzaldehyde (DEAB) to establish the negative control gate. Fluorescence signals were analyzed on a DxFLEX flow cytometer (Beckman Coulter), and the ALDH^bright^ population was quantified based on gating relative to the DEAB control.

### 2.13 Chromatin immunoprecipitation–quantitative PCR (ChIP-qPCR)

ChIP–qPCR was conducted using a commercial assay kit (Millipore, Billerica, MA, USA) according to the manufacturer's protocol. Cells were fixed with 1% formaldehyde to crosslink DNA and proteins, lysed, and subjected to sonication to shear chromatin. The fragmented chromatin was incubated overnight at 4 °C with an anti-H3K27ac antibody or control IgG. Immunoprecipitated DNA was purified and analyzed by quantitative PCR. Primer sequences used for qPCR are available upon request.

### 2.14 Co-immunoprecipitation (Co-IP) assay

Protein lysates from treated cells were incubated with specific primary antibodies or control IgG at 4 °C overnight, followed by incubation with magnetic beads (EpiZyme) for 8 h. After washing, immunoprecipitated proteins were eluted with SDS sample buffer and analyzed by Western blotting.

### 2.15 CHX chase assay

Cells with indicated modifications were treated with 100 μg/mL CHX to inhibit protein synthesis. Total protein lysates were collected at specified time points and analyzed by Western blotting to assess the degradation rate and estimate the half-life of PFKP.

### 2.16 ELISA

Total and active TGF-*β* levels were determined using a human TGF-*β*1 DuoSet (R&D Systems Europe, Abingdon, UK) and a substrate reagent pack, in accordance with the manufacturer's guidelines. Acetyl-CoA levels were assessed using an acetyl-CoA assay kit (Biovision, Milpitas, CA, USA), following the manufacturer's instructions.

### 2.17 Caspase-3 activity assay

Caspase-3 activity was assessed using a colorimetric assay kit (Abcam) according to the manufacturer's protocol. Equal amounts of protein from indicated treated cells were incubated with the caspase-3-specific substrate Ac-DEVD-pNA at 37 °C for 1–2 h. The release of pNA was quantified by measuring absorbance at 405 nm using a microplate reader. Caspase-3 activity was expressed as optical density values and compared across treatment groups.

### 2.18 Immunofluorescence

SCC-1 and SCC-23 cells cultured on glass coverslips were fixed with 4% paraformaldehyde and permeabilized with 0.5% Triton X-100. After PBS washes, cells were blocked for 1 h in blocking buffer and incubated overnight at 4 °C with primary antibodies. Subsequently, cells were washed and incubated with Alexa Fluor-conjugated secondary antibodies (Proteintech) for 1 h at room temperature, followed by DAPI staining. Images were acquired using a Leica STELLARIS 5 confocal laser scanning microscope (Leica Microsystems, Wetzlar, Germany). Fluorescence intensity profiles were generated using the microscope's built-in analysis software to assess spatial distribution and colocalization of target proteins.

### 2.19 Immunohistochemistry analysis

Both tissue microarray (TMA) sections and conventional formalin-fixed, paraffin-embedded (FFPE) whole tissue sections were used for immunohistochemistry. After deparaffinization and rehydration through a graded ethanol series, sections were blocked with goat serum for 2 h at room temperature and incubated with primary antibodies overnight at 4 °C. Following PBS washes, sections were incubated with horseradish peroxidase–conjugated secondary antibodies for 1 h at 25 °C. Signal was visualized using a 3,3′-diaminobenzidine substrate kit. For quantitative assessment, H-scores were calculated based on both staining intensity and the percentage of positively stained cells. Staining intensity was classified as 0 (negative), 1 (weak), 2 (moderate), or 3 (strong). The H-score was then calculated using Eq. (1):

(1) H−score=(1×%ofweaklystainedcells)+(2×%ofmoderatelystainedcells)+(3×%ofstronglystainedcells)

yielding a total score ranging from 0 to 300.

### 2.20 RNA sequencing

RNA-seq analysis was performed on HNSCC cells subjected to PLOD1 depletion or control treatment. Sequencing was conducted using the BGISEQ-500 platform at the Beijing Genomics Institute (Beijing, China). Reads were aligned to the human reference genome using the HISAT pipeline (http://www.ccb.jhu.edu/software/hisat/). Clean reads were mapped to reference genes with Bowtie2, and gene expression levels were quantified using the RNA-Seq by Expectation-Maximization algorithm. Differentially expressed genes (DEGs) were identified using DEGseq, with significance defined as an absolute fold change ≥2 and adjusted *P* value ≤ 0.05. GSEA was performed to identify significantly enriched pathways.

### 2.21 Targeted metabolomics

Cell pellets were extracted with pre-chilled methanol: acetonitrile: water (2:2:1, *v*/*v*/*v*) containing isotope-labeled internal standards. After sonication on ice and incubation at −80 °C, samples were centrifuged twice at 4 °C, and supernatants were collected for LC–MS/MS analysis. Chromatographic separation was performed on a BEH Amide column, and detection was carried out on an Agilent 6545 Q-TOF mass spectrometer (Agilent Technologies, Santa Clara, CA, USA) operating in both positive and negative ion modes. Raw data were processed by imputing values below the quantification limit as zero. Differential metabolites were identified using Student's *t*-test with a significance threshold of *P* < 0.05.

### 2.22 Glucose uptake and lactate production assays

For glucose uptake analysis, cells were first incubated in glucose- and serum-free medium for 6 h, followed by treatment with glucose-free DMEM containing 10 μmol/L 2-deoxy-2-[(7-nitro-2,1,3-benzoxadiazol-4-yl)amino]-d-glucose for 2 h. After washing twice with PBS, cells were analyzed by flow cytometry (BD Biosciences, Franklin Lakes, NJ, USA). Lactate production was measured using a colorimetric lactate assay kit (BioVision, Milpitas, CA, USA) according to the manufacturer's instructions. The net lactate production was calculated by subtracting the background lactate concentration in blank media from that of the experimental samples.

### 2.23 Seahorse metabolic flux analysis

The extracellular acidification rate (ECAR) and oxygen consumption rate (OCR) were measured using the Seahorse XF Analyzer (Agilent Technologies) to evaluate glycolytic and mitochondrial function. Cells were seeded in XF microplates and cultured overnight. On the day of assay, cells were washed and incubated in Seahorse assay medium under non-CO_2_ conditions. After equilibration, metabolic stress reagents were sequentially injected through the sensor cartridge, and ECAR and OCR values were recorded in real time using the manufacturer's protocol.

### 2.24 Molecular docking

The amino acid sequences of PFKP and PLOD1 were retrieved from UniProt, and their three-dimensional structures were predicted using SWISS-MODEL based on high-identity templates. Model quality was assessed using QMEAN and considered suitable for docking analysis. Protein–protein docking was performed using the HDOCK server to evaluate potential interactions between PFKP and PLOD1. The top-ranked complex based on docking score was selected for structural analysis and visualization using PyMOL (version 2.6.1).

### 2.25 *In vivo* limiting dilution assay

To evaluate the effect of PLOD1 depletion on the tumor-initiating capacity of cancer cells, primary HNSCC cells derived from PDX #1 and PDX #6 were transduced with control or PLOD1-targeting sgRNAs and injected subcutaneously into the flanks of NSG mice at limiting dilutions (10^6^, 10^5^, 10^4^, or 10^3^ cells per injection). Tumor formation was monitored over time, and the presence of palpable or visible tumors was recorded as a positive outcome. The frequency of tumor-initiating cells was calculated using Extreme Limiting Dilution Analysis (http://bioinf.wehi.edu.au/software/elda/).

### 2.26 Development of cisplatin-resistant PDX models and evaluation of PLOD1-targeted therapy

All animal experiments were performed in accordance with protocols approved by the Institutional Animal Care and Ethics Committee (approval No. SMUL202405016). Mice were obtained from the Guangdong Medical Laboratory Animal Center (Foshan, Guangdong, China). To identify metabolic enzymes associated with cisplatin resistance, fresh surgical specimens from patients with HNSCC were processed into 1–3 mm^3^ fragments and subcutaneously implanted into the flanks of NOD/SCID/IL2r*γ*^null^ (NSG) mice. Tumor growth was monitored every three days. Once tumors reached ∼100 mm^3^, mice were treated with cisplatin (5 mg/kg, intraperitoneally, once weekly) or vehicle. Tumors showing regression were classified as cisplatin-sensitive, while those exhibiting regrowth after an initial response were defined as cisplatin-resistant. Tumor tissues were collected for downstream analyses to identify candidate resistance-associated enzymes.

To assess the therapeutic relevance of targeting PLOD1, PDX-bearing mice were randomized when tumor volume reached ∼50 mm^3^ and treated with LNP-formulated siRNA against PLOD1 (LNP-siPLOD1), cisplatin, or the combination. LNP-siRNAs were administered intravenously, and cisplatin was given intraperitoneally. Tumor growth was monitored every three days, and tumors were harvested at endpoint for analysis.

### 2.27 4-NQO-induced oral carcinogenesis model

To establish an immunocompetent model for evaluating cisplatin response, six-week-old C57BL/6 mice were administered 4-nitroquinoline-1-oxide (4-NQO; 50 μg/mL) in drinking water for 16 weeks to induce oral tumorigenesis, followed by regular water for an additional 10 weeks. At week 26, mice bearing oral tumors of comparable size were enrolled and treated with cisplatin (5 mg/kg, intraperitoneally, once weekly). Tumor response was monitored, and mice were stratified into cisplatin-sensitive or cisplatin-resistant groups based on treatment outcomes. Tumor tissues were collected for downstream evaluation of PLOD1 expression.

### 2.28 Subcutaneous tumor model

To establish a subcutaneous xenograft model, 2 × 10^6^ treated HNSCC cells were injected into the dorsal flanks of six-week-old male BALB/c nude mice. Mice were randomly assigned to different experimental groups. Tumor growth and body weight were monitored throughout the study. Four weeks after injection, mice were euthanized, and tumors were excised for volume and weight measurements.

### 2.29 Orthotopic tongue cancer model and lymph node analysis

To establish an orthotopic tongue cancer model, 2 × 10^5^ SCC-1 cells suspended in 20 μL of serum-free medium were injected into the anterior tongue of six-week-old immunodeficient mice under anesthesia. Once tumors were established, mice were randomized into four treatment groups: LNP-siCTRL, LNP-siPLOD1, cisplatin, and LNP-siPLOD1 plus cisplatin. LNP-formulated siRNAs were administered intravenously, and cisplatin was delivered intraperitoneally at 5 mg/kg once weekly. At the experimental endpoint, cervical lymph nodes were collected, fixed, paraffin-embedded, and sectioned. Immunohistochemical staining was performed using an anti-pan-cytokeratin antibody to detect metastatic cancer cells.

### 2.30 Statistical analysis

All statistical analyses were conducted using GraphPad Prism version 9.0 (GraphPad Software, San Diego, CA, USA). Data are presented as mean ± standard deviation (SD). Comparisons between two groups were performed using unpaired two-tailed Student's *t*-tests, and one-way ANOVA was used for multiple group comparisons. Survival curves were generated using the Kaplan–Meier method and compared using the log-rank test. Correlations between variables were evaluated using Spearman's rank correlation coefficient. Statistical significance was defined as *P* < 0.05.

## 3 Results

### 3.1 Elevated PLOD1 confers cisplatin resistance and predicts poor prognosis in HNSCC

**Figure 1:**
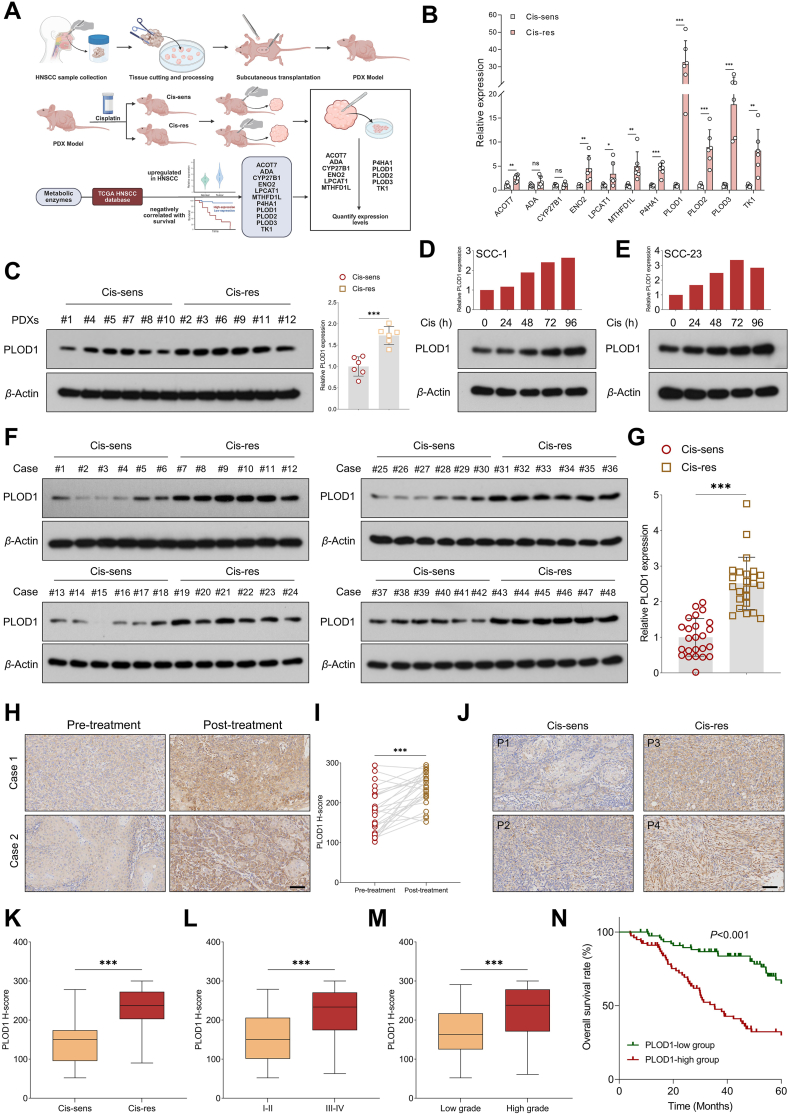
PLOD1 is upregulated in cisplatin-resistant HNSCC and associated with poor prognosis. (A) Schematic representation of the workflow. Fresh HNSCC tumor samples were processed and subcutaneously implanted into immunodeficient mice to generate PDX models. Following cisplatin treatment, tumors were classified as cisplatin-sensitive (*Cis*-sens) or cisplatin-resistant (*Cis*-res). Candidate metabolic enzymes were identified based on TCGA expression and survival data, and subsequently validated in tumors. (B) Relative mRNA expression levels of candidate metabolic enzymes in *Cis*-sens (*n* = 6) and *Cis*-res (*n* = 6) PDX tumors as determined by quantitative PCR. (C) Immunoblotting of PLOD1 in representative *Cis*-sens (*n* = 6) and *Cis*-res (*n* = 6) PDXs with corresponding quantification. (D, E) Time-course immunoblot analysis of PLOD1 expression in SCC-1 and SCC-23 cells following cisplatin treatment (*n* = 3). (F, G) PLOD1 protein levels in primary HNSCC tumors classified by cisplatin response across 48 patient samples. (H, I) Representative IHC images showing PLOD1 expression in paired HNSCC tumor samples before and after cisplatin treatment, and corresponding quantification of PLOD1 H-scores. Scale bar, 100 μm. (J, K) Representative IHC images of PLOD1 expression in tumors from *Cis*-sens and *Cis*-res HNSCC patients, along with corresponding H-score comparison between the two groups. Scale bar, 100 μm. (L, M) PLOD1 H-score distribution in HNSCC tissues stratified by clinical stage and histological grade. (N) Kaplan–Meier survival curves for overall survival in an in-house HNSCC cohort, categorized by high and low PLOD1 expression. Data are presented as mean ± SD unless otherwise indicated. Two-group comparisons were performed using two-tailed Student's *t*-test. ∗*P* < 0.05, ∗∗*P* < 0.01, ∗∗∗*P* < 0.001; ns, not significant.

To identify key metabolic enzymes driving cisplatin resistance in HNSCC, PDX models were first established by implanting tumor tissues from HNSCC patients into immunodeficient mice. After tumor engraftment, mice were treated with cisplatin and tumors were classified as cisplatin-sensitive (*Cis*-sens) or cisplatin-resistant (*Cis*-res) based on their growth response. Next, using bioinformatics analysis of TCGA HNSCC cohort, we screened metabolic enzymes significantly upregulated in tumors compared with normal tissues and negatively correlated with patient survival. Eleven candidates met these criteria: *ACOT7*, *ADA*, *CYP27B1*, *ENO2*, *LPCAT1*, *MTHFD1L*, *P4HA1*, *PLOD1*, *PLOD2*, *PLOD3*, and *TK1* (Fig. 1A). Finally, we validated these candidates using qPCR to compare their expression levels in *Cis*-sens and *Cis*-res PDX tumors. Among these, *PLOD1* demonstrated the most pronounced upregulation in *Cis*-res tumors, implicating PLOD1 as a key metabolic contributor to cisplatin resistance and poor clinical outcomes in HNSCC (Fig. 1B). Consistently, PLOD1 protein levels were significantly elevated in *Cis*-res PDX tumors compared with *Cis*-sens counterparts (Fig. 1C). To validate these findings in an immunocompetent setting, we employed a 4NQO-induced oral carcinogenesis mouse model followed by cisplatin treatment to generate *Cis*-sens and *Cis*-res tumors (Supporting Information Fig. S1A). WB analysis revealed significantly elevated PLOD1 protein levels in *Cis*-res tumors compared to their sensitive counterparts (Fig. S1B).

These *in vivo* observations prompted us to investigate whether PLOD1 upregulation reflects a broader feature of HNSCC biology. PLOD1 expression is consistently higher in HNSCC cells compared with normal epithelial cells. Moreover, its expression pattern parallels the known aggressiveness of these lines: UM-1 expresses higher levels than UM-2, and UMSCC-5 expresses higher levels than UMSCC-6 (Fig. S1C). To determine whether PLOD1 is also dynamically regulated by therapeutic pressure, we examined its response to cisplatin treatment. *In vitro* experiments revealed that cisplatin treatment induced a time-dependent increase in PLOD1 expression in both SCC-1 and SCC-23 cell lines (Fig. 1D and E**)**. To validate the clinical relevance of PLOD1 upregulation, we analyzed PLOD1 protein expression in tumor samples from HNSCC patients with known cisplatin response status. WB analysis of 48 clinical tumor specimens revealed that PLOD1 protein levels were significantly higher in cisplatin-resistant tumors compared to cisplatin-sensitive ones (Fig. 1F and G). Furthermore, in paired tumor samples collected from patients before and after cisplatin-based treatment, IHC staining demonstrated a marked increase in PLOD1 staining intensity following therapy (Fig. 1H and I), indicating a potential treatment-induced upregulation. Consistently, resistant tumors exhibited stronger PLOD1 staining than sensitive tumors in an independent patient cohort (Fig. 1J and K), reinforcing the association between elevated PLOD1 levels and cisplatin resistance in clinical HNSCC samples.

To further explore the clinical relevance of PLOD1, we next examined its expression patterns in tumor and normal tissues across multiple public datasets. In the TCGA HNSCC cohort and GEO datasets including GSE127165, GSE6631, GSE37991, and GSE58911, PLOD1 expression was significantly elevated in tumor tissues compared with matched adjacent normal tissues (ANTs) (Supporting Information Fig. S2A–S2F). Similarly, analysis of datasets GSE25099, GSE34105, GSE143224 and GSE31056 confirmed higher *PLOD1* expression in tumor samples relative to independent normal tissues (Fig. S2G–S2J). Notably, dataset GSE30784 showed a stepwise upregulation of PLOD1 from normal epithelium to dysplastic lesions and cancer (Fig. S2K), suggesting a potential role for PLOD1 in HNSCC initiation and progression. Consistently, high *PLOD1* expression was associated with significantly poorer overall survival in the TCGA HNSCC, GSE65858, and GSE41613 cohorts (Fig. S2L–S2N). To investigate whether the prognostic value of PLOD1 extends beyond HNSCC, we performed pan-cancer survival analyses across TCGA cohorts. High *PLOD1* expression was significantly associated with poorer overall survival in multiple cancer types, including CESC, CHOL, ESCA, GBM, KIRC, KIRP, LAML, LGG, LIHC, LUAD, MESO, OV, PAAD, SARC, STAD, and UCS (Supporting Information Fig. S3A–S3P). In line with the above bioinformatic findings, our in-house cohort demonstrated that PLOD1 expression was significantly higher in advanced-stage tumors compared to early-stage tumors (Fig. 1L), and in high-grade tumors relative to low-grade counterparts (Fig. 1M). Importantly, patients with high PLOD1 expression exhibited significantly poorer overall survival than those with low expression (Fig. 1N). Collectively, these findings indicate that elevated PLOD1 promotes cisplatin resistance and, consequently, poorer patient outcomes, warranting further mechanistic investigation.

### 3.2 PLOD1 depletion sensitizes HNSCC cells to cisplatin

**Figure 2:**
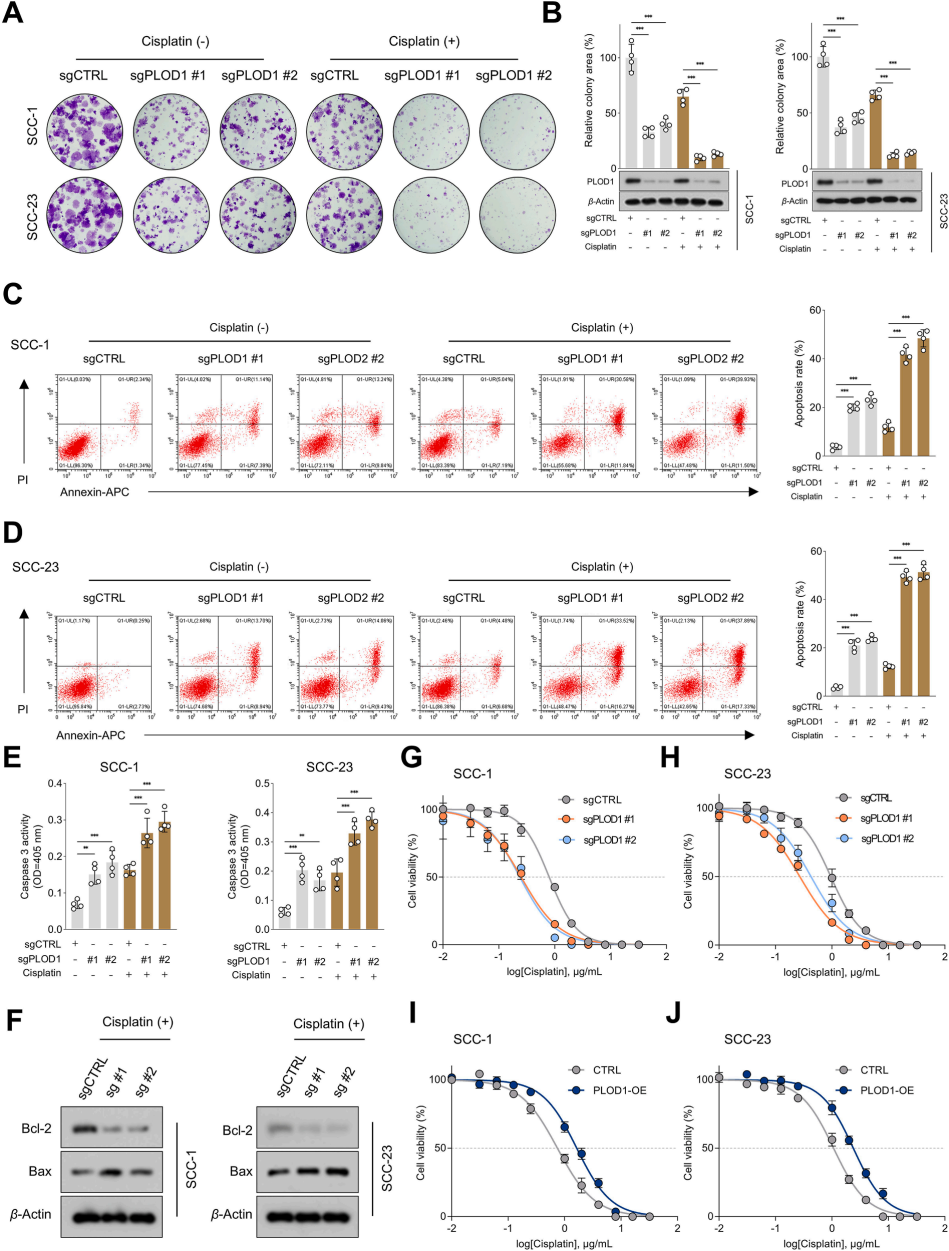
PLOD1 depletion sensitizes HNSCC cells to cisplatin treatment. (A, B) Colony formation assays were performed in SCC-1 and SCC-23 cells transduced with control (sgCTRL) or PLOD1-targeting (sgPLOD1 #1 and #2) short guide RNAs, followed by treatment with or without cisplatin (*n* = 4). (C, D) Apoptotic cell populations in SCC-1 and SCC-23 cells transduced with sgCTRL, sgPLOD1 #1, or sgPLOD1 #2 were assessed by flow cytometry, both with and without cisplatin treatment (*n* = 4). (E) Caspase-3 activity was measured in SCC-1 and SCC-23 cells with PLOD1 depletion, with or without cisplatin treatment (*n* = 4). (F) Western blot analysis of the apoptosis-related proteins Bcl-2 and Bax in SCC-1 and SCC-23 cells transduced with sgCTRL or sgPLOD1, following cisplatin exposure (*n* = 3). (G–J) Cell viability of SCC-1 and SCC-23 cells with PLOD1 depletion or overexpression following treatment with increasing concentrations of cisplatin. IC_50_ values for SCC-1 (0.80 μg/mL) and SCC-23 (0.95 μg/mL) are indicated for reference (*n* = 3). Data are presented as mean ± SD unless otherwise indicated. Two-group comparisons were performed using two-tailed unpaired Student's *t*-test. For comparisons among multiple groups, one-way ANOVA with *post hoc* multiple-comparisons testing was used. ∗∗*P* < 0.01, ∗∗∗*P* < 0.001.

Given the clinical association between PLOD1 upregulation and cisplatin resistance, we next investigated whether PLOD1 depletion could enhance cisplatin sensitivity in HNSCC cells. CRISPR/Cas9-mediated knockout of PLOD1 (sgPLOD1 #1/#2) partially reduced clonogenic growth in SCC-1 and SCC-23 cells and, when combined with cisplatin, led to a pronounced additional reduction in colony formation (Fig. 2A and B). Consistently, Annexin V/PI staining revealed that PLOD1 loss markedly enhanced cisplatin-induced apoptosis, whereas either intervention alone produced only moderate effects (Fig. 2C and D). To further elucidate the pro-survival role of PLOD1 in the context of cisplatin exposure, we assessed apoptosis-related markers in PLOD1-deficient cells. Caspase-3 activity was significantly increased in sgPLOD1-transduced SCC-1 and SCC-23 cells upon cisplatin treatment compared to controls (Fig. 2E). In line with this, WB analysis showed reduced Bcl-2 and elevated Bax expression in PLOD1-knockout cells following cisplatin exposure (Fig. 2F), indicating activation of apoptotic signaling. Moreover, dose-response analyses demonstrated that PLOD1 loss sensitized cells to cisplatin, as reflected by leftward shifts in IC_50_ values in both SCC-1 and SCC-23 cells (Fig. 2G and H). Conversely, enforced expression of PLOD1 conferred partial resistance to cisplatin, as indicated by rightward shifts in the dose–response curves (Fig. 2I and J). Together, these results further support a functional role for PLOD1 in promoting cisplatin resistance in HNSCC.

### 3.3 PLOD1 promotes cancer stem-like properties and tumor-initiating capacity *in vitro* and *in vivo*

**Figure 3:**
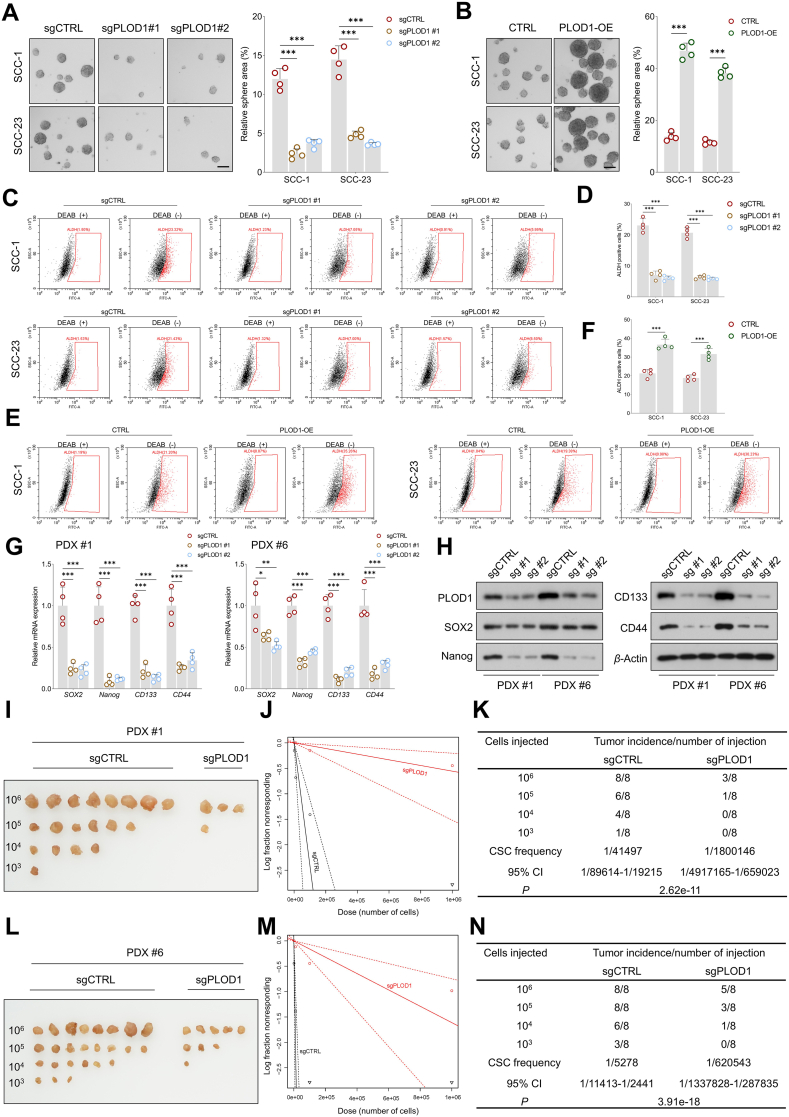
PLOD1 drives cancer stem-like properties and tumor-initiating capacity *in vitro* and *in vivo*. (A, B) Sphere formation assays were conducted in SCC-1 and SCC-23 cells with PLOD1 knockout or overexpression to assess their capacity for stem-like sphere formation (*n* = 4). (C, D) ALDH expression was assessed by flow cytometry in SCC-1 and SCC-23 cells with PLOD1 knockout or control, with 15 μmol/L DEAB treatment for 30 min included as the negative control (*n* = 4). (E, F) ALDH expression analysis in SCC-1 and SCC-23 cells with PLOD1 overexpression or control, with 15 μmol/L DEAB treatment for 30 min included as the negative control (*n* = 4). (G, H) mRNA and protein expression levels of SOX2, Nanog, CD133, and CD44 were analyzed by qRT-PCR and Western blot in primary HNSCC cells isolated from PDX #1 and PDX #6 tumors following PLOD1 depletion (*n* = 4). (I–N) *In vivo* limiting dilution assays were performed using HNSCC cells derived from PDX #1 or PDX #6 tumors, with PLOD1 knockout in the cells, to assess tumor formation at decreasing cell doses (*n* = 8 per dose). CSC frequency was determined using ELDA. Data are presented as mean ± SD unless otherwise indicated. Two-group comparisons were performed using two-tailed unpaired Student's *t*-test. For comparisons among multiple groups, one-way ANOVA with *post hoc* multiple-comparisons testing was used. ∗*P* < 0.05, ∗∗*P* < 0.01, ∗∗∗*P* < 0.001.

Cancer stemness has been implicated as a key driver of cisplatin resistance in various malignancies, including HNSCC^18^, ^19^, ^20^. To investigate whether PLOD1 contributes to chemoresistance by promoting stem-like properties, we assessed tumorsphere formation and ALDH activity following PLOD1 modulation. PLOD1 depletion significantly reduced sphere-forming capacity in SCC-1 and SCC-23 cells, whereas PLOD1 overexpression enhanced sphere growth (Fig. 3A and B). Consistently, the proportion of ALDH-positive cells was markedly decreased in PLOD1-knockdown cells and increased upon PLOD1 overexpression (Fig. 3C–F). In line with these flow cytometry results, immunoblotting confirmed a concordant reduction of ALDH1A1 protein levels following PLOD1 silencing and an increase upon PLOD1 overexpression (Supporting Information Fig. S4A and S4B). To determine whether the stemness-regulatory function of PLOD1 extends *in vivo*, we examined CSC markers and tumor-initiating capacity in PDX models following PLOD1 depletion. RT-qPCR and WB analyses showed that PLOD1 knockdown in two independently derived PDX lines—PDX #1 (cisplatin-sensitive) and PDX #6 (cisplatin-resistant)—significantly reduced the expression of core stemness regulators including SOX2, Nanog, CD133, and CD44 (Fig. 3G and H). Limiting dilution assays further demonstrated that PLOD1-deficient tumor cells exhibited substantially reduced tumor-initiating capacity in immunodeficient mice (Fig. 3I–N). These results indicate that PLOD1 supports cancer stemness both *in vitro* and *in vivo*, reinforcing its potential role in driving chemoresistance.

### 3.4 PLOD1 sustains EMT through TGFBR2–TGF-β signaling and preserves stemness *via* alternative pathways in cisplatin-treated HNSCC

**Figure 4:**
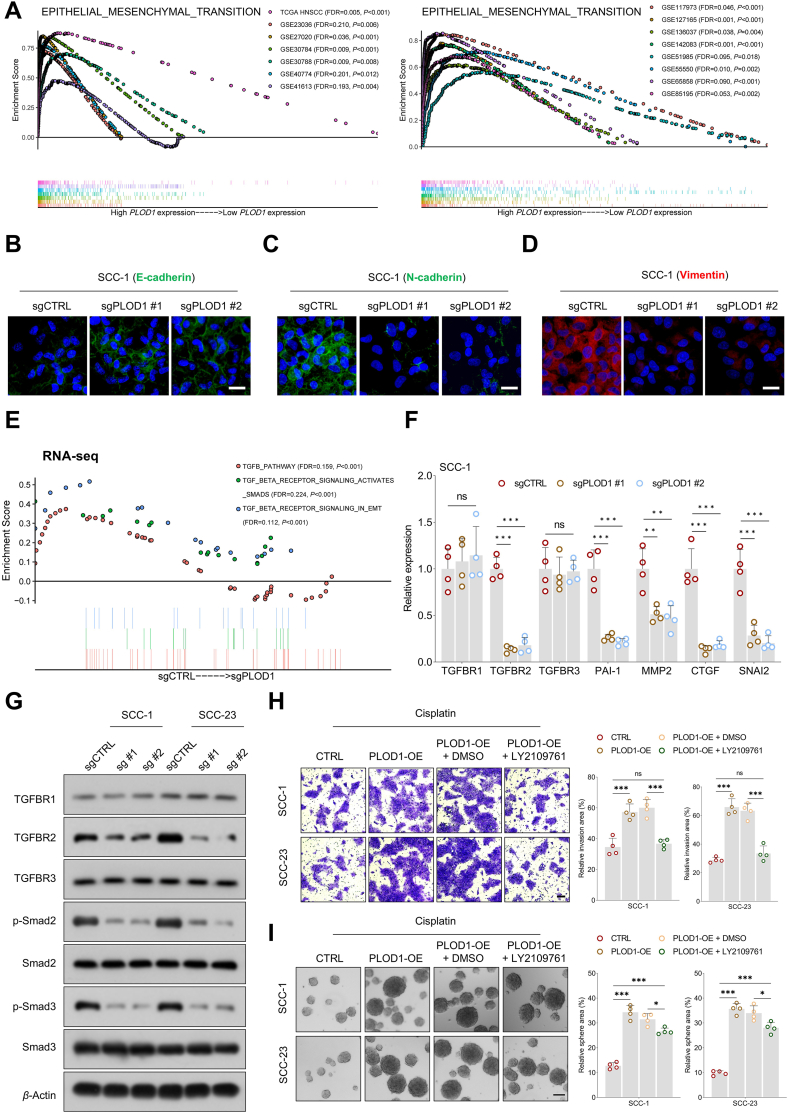
PLOD1 modulates EMT and TGF-*β* pathway activation in HNSCC. (A) GSEA plots showing the enrichment of EMT-related pathways in HNSCC datasets from TCGA and other publicly available datasets, comparing high and low *PLOD1* expression groups. Datasets are listed with corresponding FDR values and enrichment scores. (B–D) Immunofluorescence staining of SCC-1 cells for epithelial (E-cadherin) and mesenchymal (N-cadherin and vimentin) markers following PLOD1 depletion. Scale bar, 100 μm. (E) GSEA analysis of RNA-seq data from SCC-1 cells with PLOD1 knockout or control, illustrating the enrichment of TGF-*β*-related pathways in the control group. (F) Quantification of mRNA expression levels of *TGFBR1*, *TGFBR2*, *TGFBR3*, *PAI-1*, *MMP2*, *CTGF*, and *SNAI2* in SCC-1 cells with PLOD1 knockout, as determined by qRT-PCR (*n* = 4). (G) Western blot analysis of TGFBR1, TGFBR2, TGFBR3, *p*-Smad2, Smad2, *p*-Smad3, and Smad3 in SCC-1 and SCC-23 cells with PLOD1 knockout (*n* = 3). (H) Transwell invasion assays showing the invasive capacity of SCC-1 and SCC-23 cells with PLOD1 overexpression in the presence or absence of the TGF-*β* receptor inhibitor LY2109761 (10 μmol/L), following cisplatin treatment (*n* = 4). Scale bar, 100 μm. (I) Tumor sphere formation assays in SCC-1 and SCC-23 cells with PLOD1 overexpression, treated with or without LY2109761 (10 μmol/L) following cisplatin exposure (*n* = 4). Data are presented as mean ± SD unless otherwise indicated. For comparisons among multiple groups, one-way ANOVA with *post hoc* multiple-comparisons testing was used. ∗*P* < 0.05, ∗∗*P* < 0.01, ∗∗∗*P* < 0.001; ns, not significant.

To elucidate the molecular mechanisms underlying PLOD1-mediated stemness-associated cisplatin resistance in HNSCC, we performed gene set enrichment analysis using TCGA HNSCC and multiple GEO datasets. Patients were stratified into high and low *PLOD1* expression groups based on the median transcript level. EMT emerged as the most consistently and significantly enriched pathway in the high *PLOD1* group across cohorts (Fig. 4A), suggesting a potential link between PLOD1 expression and epithelial–mesenchymal plasticity. To further explore this association, we performed correlation analyses in the TCGA HNSCC cohort. *PLOD1* expression was negatively correlated with epithelial markers such as *CDH1* and *TJP1*, and positively correlated with a broad panel of mesenchymal markers and EMT transcription factors, including *CDH2*, *VIM*, *SNAI1*, *ZEB1*, *ZEB2*, and *TWIST1* (Fig. S4C and S4D). To validate this relationship experimentally, we examined EMT marker expression in HNSCC cells following PLOD1 depletion. Immunofluorescence staining revealed increased expression of the epithelial marker E-cadherin and decreased expression of the mesenchymal markers N-cadherin and vimentin upon PLOD1 knockout (Fig. 4B–D, Fig. S4E–S4G).

Transcriptomic analysis by RNA-seq revealed that PLOD1 depletion leads to a broad suppression of TGF-*β* signaling, as indicated by the downregulation of TGFB_PATHWAY, SMAD activation, and EMT-related gene sets, implicating this axis in PLOD1-driven stemness and drug resistance (Fig. 4E). However, based on the ELISA results shown in Fig. S4H–S4K, neither the active nor total levels of TGF-*β* were significantly altered upon PLOD1 knockout or overexpression in both SCC-1 and SCC-23 cell lines. We hypothesized that the attenuated TGF-*β* signaling observed in RNA-seq might result from alterations in receptor expression rather than ligand availability. To test this, we assessed the transcriptional and protein levels of TGF-*β* receptors and downstream effectors. qRT-PCR revealed a specific downregulation of *TGFBR2*, while *TGFBR1* and *TGFBR3* remained unaffected. This was accompanied by reduced expression of canonical TGF-*β* target genes, including *PAI-1*, *MMP2*, *CTGF*, and *SNAI2* (Fig. 4F and Supporting Information Fig. S5A). Conversely, PLOD1 overexpression led to a pronounced upregulation of *TGFBR2* and the same downstream targets, without affecting *TGFBR1* or *TGFBR3* (Fig. S5B and S5C). Immunoblotting confirmed a decrease in TGFBR2 protein and reduced phosphorylation of Smad2 and Smad3, with total Smad levels unchanged (Fig. 4G and Fig. S5D). These results suggest that PLOD1 sustains TGF-*β* pathway activity by maintaining TGFBR2 expression.

To assess the functional contribution of the PLOD1–TGF-*β* axis under cisplatin treatment, we exposed PLOD1-overexpressing SCC-1 and SCC-23 cells to the TGF-*β* receptor inhibitor LY2109761. Inhibition of TGF-*β* signaling completely abrogated the PLOD1-induced EMT phenotype, restoring cell invasiveness to baseline levels (Fig. 4H). In contrast, LY2109761 only partially suppressed the enhanced clonogenicity and tumoursphere formation, both of which remained significantly elevated relative to control (Fig. 4I, Fig. S5E and S5F). Immunoblotting confirmed that LY2109761 reversed the mesenchymal shift induced by PLOD1 overexpression, as indicated by reduced N-cadherin and vimentin and increased E-cadherin levels. However, stemness markers such as SOX2, CD44, and CD133 remained upregulated despite TGF-*β* inhibition (Fig. S5G and S5H). These findings suggest that while the EMT program driven by PLOD1 is predominantly TGF-*β*-dependent, the regulation of stemness involves additional, TGF-*β*-independent mechanisms.

### 3.5 PLOD1 drives glycolysis and malignant phenotypes in HNSCC in a PFKP-dependent manner

**Figure 5:**
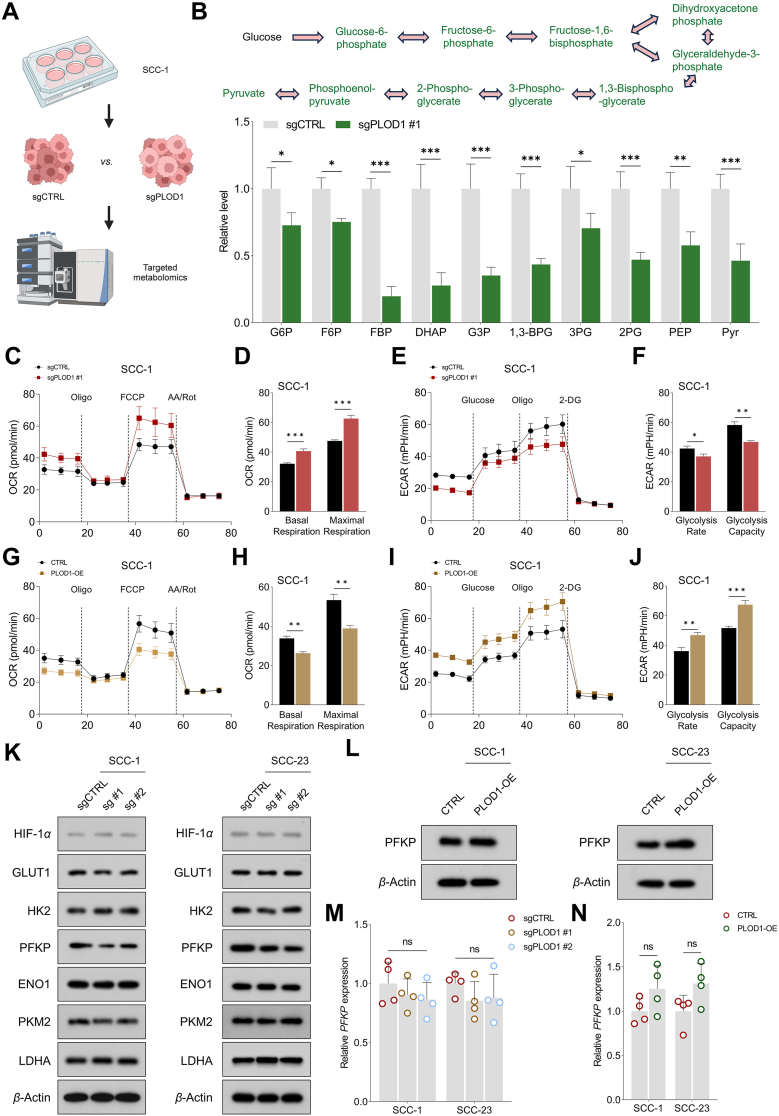
PLOD1 drives glycolysis and regulates PFKP expression in HNSCC. (A) Schematic overview of experimental design comparing SCC-1 cells with PLOD1 depletion versus control, followed by targeted metabolomics analysis. (B) Relative levels of key metabolites in the glycolytic pathway, as measured by targeted metabolomics in SCC-1 cells with PLOD1 depletion. (C, D) OCR analysis and quantification of basal and maximal respiration rates in SCC-1 cells with PLOD1 depletion under basal conditions, following FCCP stimulation, and after AA/Rot treatment (*n* = 3). (E, F) ECAR analysis and quantification of glycolysis rate and glycolysis capacity in SCC-1 cells with PLOD1 depletion under glucose, oligomycin, and 2-DG treatments (*n* = 3). (G, H) OCR analysis and quantification of basal and maximal respiration rates in SCC-1 cells with PLOD1 overexpression (*n* = 3). (I, J) ECAR analysis and quantification of glycolysis rate and glycolysis capacity in SCC-1 cells with PLOD1 overexpression (*n* = 3). (K) Western blot analysis of HIF-1*α*, GLUT1, HK2, PFKP, ENO1, PKM2, and LDHA in SCC-1 and SCC-23 cells with PLOD1 depletion (*n* = 3). (L) Western blot analysis of PFKP expression in SCC-1 and SCC-23 cells with PLOD1 overexpression (*n* = 3). (M, N) Quantification of relative *PFKP* mRNA expression in SCC-1 and SCC-23 cells with PLOD1 depletion or overexpression (*n* = 4). Data are presented as mean ± SD unless otherwise indicated. Two-group comparisons were performed using two-tailed unpaired Student's *t*-test. For comparisons among multiple groups, one-way ANOVA with *post hoc* multiple-comparisons testing was used. ∗*P* < 0.05, ∗∗*P* < 0.01, ∗∗∗*P* < 0.001; ns, not significant.

Given that PLOD1 is an enzyme implicated in metabolic regulation, we performed targeted metabolomic profiling to investigate its role in cellular energy metabolism^21^^,^^22^. LC–MS/MS analysis of SCC-1 cells revealed that PLOD1 knockout led to a widespread decrease in glycolytic intermediates, including glucose-6-phosphate (G6P), fructose-6-phosphate (F6P), fructose-1,6-bisphosphate (FBP), and downstream metabolites such as 3-phosphoglycerate (3 PG), phosphoenolpyruvate (PEP), and pyruvate (Fig. 5A and B). Consistently, glucose uptake and lactate production were significantly decreased in PLOD1-depleted SCC-1 and SCC-23 cells, whereas PLOD1 overexpression resulted in the opposite effect (Supporting Information Fig. S6A–S6H). Seahorse analysis demonstrated that PLOD1 knockout decreased ECAR while increasing both basal and maximal OCR, indicating reduced glycolytic activity accompanied by a compensatory enhancement of mitochondrial oxidative phosphorylation glycolysis **(**Fig. 5C–F, Fig. S6I–S6L). Conversely, PLOD1 overexpression increased ECAR and was associated with reduced mitochondrial respiration (Fig. 5G–J, Fig. S6M–S6P).

We next examined the expression of key glycolytic enzymes and regulators in HNSCC cells following PLOD1 depletion. PLOD1 knockout had no effect on the protein levels of GLUT1, HK2, ENO1, PKM2, LDHA, or HIF-1*α* in either SCC-1 or SCC-23 cells. In contrast, PFKP protein levels were consistently reduced in PLOD1-deficient cells and increased upon PLOD1 overexpression (Fig. 5K and L). Notably, PFKP transcript levels remained unchanged across conditions (Fig. 5M and N), suggesting that PLOD1 modulates glycolysis primarily through post-transcriptional regulation of PFKP.

To determine whether PFKP mediates the pro-tumorigenic effects of PLOD1, we performed rescue experiments using shRNA targeting PFKP in PLOD1-overexpressing cells. PFKP silencing abrogated the PLOD1-induced increases in clonogenic growth, Matrigel invasion, and tumoursphere formation in both SCC-1 and SCC-23 cells (Supporting Information Fig. S7A–S7C). Western blot analysis showed that PFKP knockdown restored E-cadherin expression and reduced mesenchymal and stemness-associated markers, including N-cadherin, vimentin, SOX2, CD44, and CD133 (Fig. S7D). Seahorse analysis further revealed that PFKP depletion markedly attenuated the ECAR increase induced by PLOD1 overexpression and simultaneously alleviated the suppression of OCR, indicating that PFKP is required for PLOD1-driven metabolic reprogramming **(**Fig. S7E–S7H). These findings collectively identify PFKP as a key downstream effector of PLOD1 in HNSCC.

### 3.6 PLOD1 protects PFKP from ubiquitin-mediated degradation *via* direct interaction

**Figure 6:**
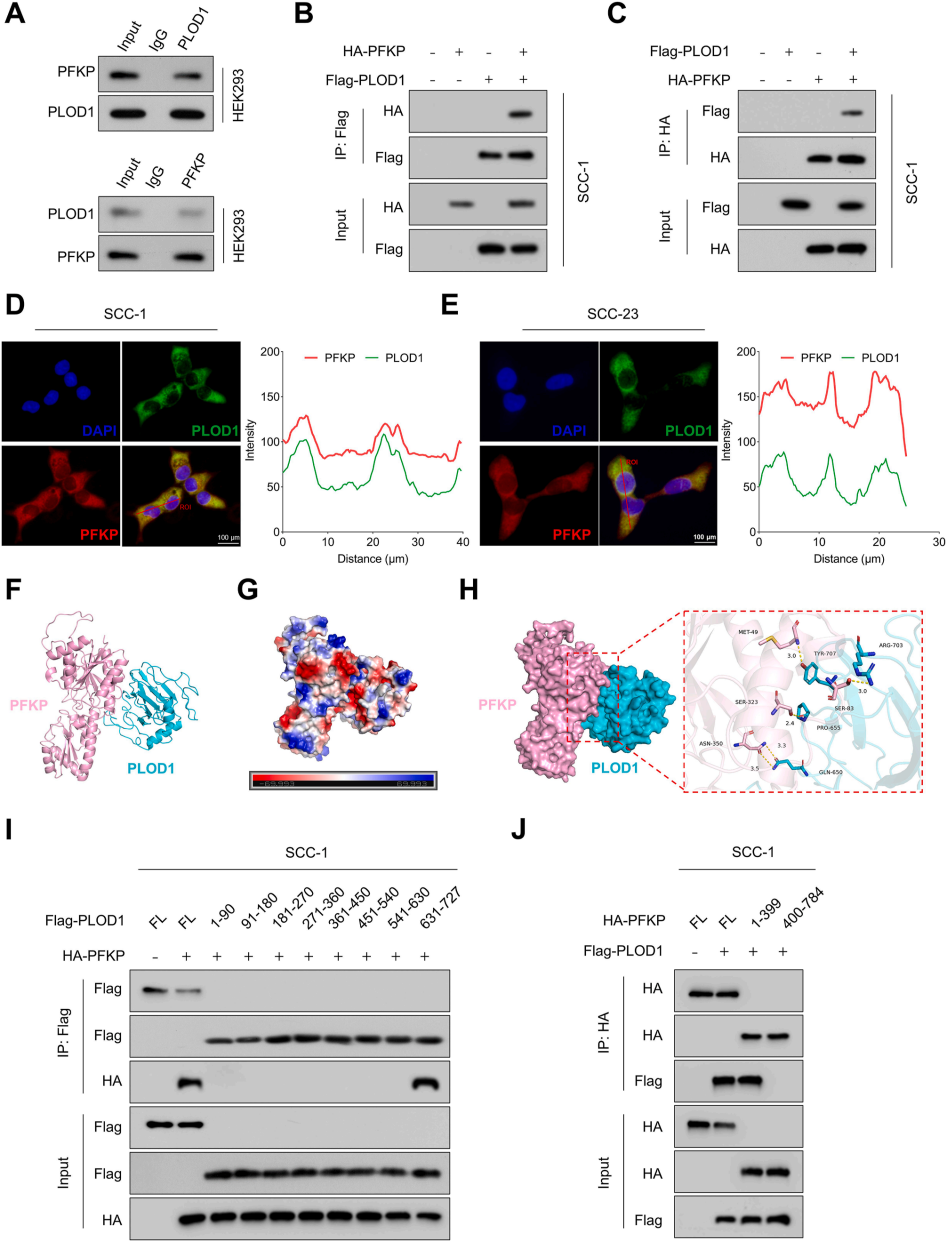
PLOD1 interacts with PFKP in HNSCC. (A) Co-IP analysis showing the interaction between endogenous PLOD1 and PFKP in HEK293 cells. Input and immunoprecipitated proteins were analyzed by Western blot (*n* = 3). (B, C) Co-IP analysis of the interaction between exogenous PLOD1 and PFKP in SCC-1 cells is shown. The interaction between Flag-tagged PLOD1 and HA-tagged PFKP was immunoprecipitated using an anti-Flag antibody, and the reverse interaction between Flag-tagged PLOD1 and HA-tagged PFKP was immunoprecipitated using an anti-HA antibody (*n* = 3). (D, E) Confocal immunofluorescence analysis in SCC-1 and SCC-23 cells showing the co-localization of PFKP and PLOD1 (*n* = 3). (F) Molecular modeling showing the interaction between PFKP and PLOD1, highlighting their potential binding interface. (G) Surface electrostatic potential map of PFKP and PLOD1, with the interaction interface shown and colored by electrostatic charge. (H) Detailed view of the PFKP–PLOD1 interface, showing key interacting residues critical for binding. (I) Co-IP analysis of truncated Flag-PLOD1 constructs in SCC-1 cells, showing that specific regions of PLOD1 interact with HA-PFKP. (J) Co-IP analysis of HA-PFKP truncations in SCC-1 cells, identifying the specific PFKP regions responsible for interacting with Flag-PLOD1 (*n* = 3).

Given that PFKP transcript levels remained unchanged while its protein abundance was significantly altered by PLOD1, we hypothesized that PLOD1 may regulate PFKP at the post-transcriptional level, potentially through a direct interaction. To test this, we performed Co-IP assays in HEK293 and SCC-1 cells. Endogenous PLOD1 and PFKP were detected in reciprocal pulldowns, confirming their association (Fig. 6A). Ectopic expression of Flag-PLOD1 and HA-PFKP further validated this interaction in HNSCC cells (Fig. 6B and C, Supporting Information Fig. S8A and S8B). Immunofluorescence staining showed cytoplasmic colocalization of PLOD1 and PFKP in both SCC-1 and SCC-23 cells, as supported by line-scan intensity analysis (Fig. 6D and E). To further characterize the interaction between PLOD1 and PFKP, we performed protein–protein docking analysis. Docking analysis predicted a potential interaction between the two proteins, and the top-ranked model originated from the largest cluster (cluster size = 100) with a high confidence score (0.9543). The predicted complex exhibited a favorable docking energy (−301.89 kcal/mol) and a sizeable buried surface area at the interface (PFKP: 1367.2 Å^2^; PLOD1: 1526.6 Å^2^), indicating a stable binding configuration (Fig. 6F). Electrostatic surface modeling further revealed complementary charge distributions at the contact region (Fig. 6G). Structural analysis of the binding interface identified multiple hydrogen bonds within distances of 2.0–3.5 Å (Fig. 6H), collectively supporting a direct and potentially stable interaction between PLOD1 and PFKP. To define the binding interface, we generated a series of PLOD1 truncation mutants. Co-IP analysis identified residues 631–727 of PLOD1 as both necessary and sufficient for binding to PFKP (Fig. 6I). Similarly, mapping analysis using PFKP fragments revealed that residues 1–399 are required for interaction with PLOD1 (Fig. 6J). These findings precisely define the interaction domains between PLOD1 and PFKP and support a direct regulatory relationship.

**Figure 7:**
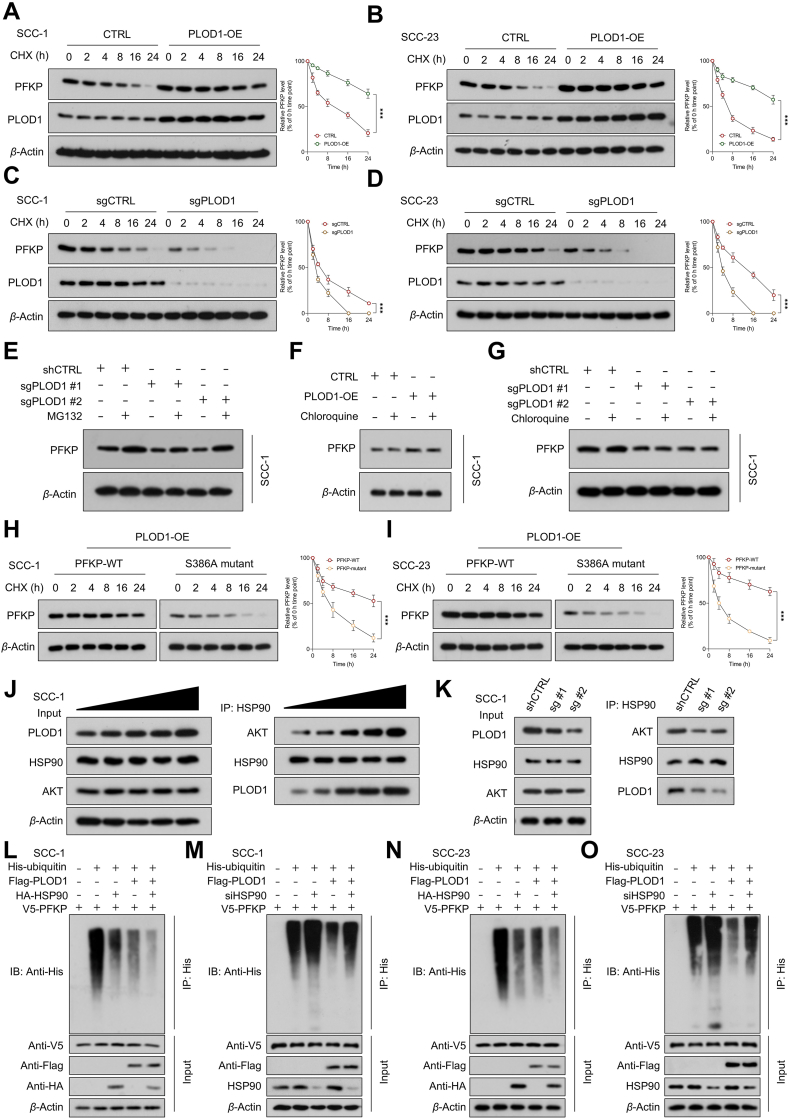
PLOD1 enhances PFKP stability through regulation of HSP90–AKT interaction and suppression of ubiquitin-mediated degradation. (A, B) CHX (100 μg/mL) chase assays in SCC-1 and SCC-23 cells, demonstrating the degradation of PFKP upon PLOD1 overexpression (*n* = 3). (C, D) PFKP turnover in SCC-1 and SCC-23 cells was assessed following PLOD1 depletion using a CHX chase assay (*n* = 3). (E) Effect of MG132 treatment on PFKP levels in SCC-1 cells with PLOD1 depletion, assessed by Western blot analysis (*n* = 3). (F, G) Assessment of changes in PFKP protein levels in SCC-1 cells with PLOD1 overexpression or depletion following chloroquine treatment, analyzed by Western blot (*n* = 3). (H, I) CHX chase assays were conducted in SCC-1 and SCC-23 cells stably overexpressing PLOD1 and transfected with either wild-type (WT) PFKP or the S386A mutant to evaluate PFKP protein stability (*n* = 3). (J) Co-IP assays were performed in SCC-1 cells using an anti-HSP90 antibody to assess the interaction between HSP90 and AKT in the presence of increasing amounts of PLOD1(*n* = 3). (K) SCC-1 cells transduced with control or PLOD1-targeting sgRNAs were subjected to co-immunoprecipitation with an anti-HSP90 antibody to assess the impact of PLOD1 loss on the HSP90–AKT interaction (*n* = 3). (L, M) PFKP ubiquitination was examined in SCC-1 cells co-transfected with the indicated constructs under HSP90 overexpression or knockdown conditions (*n* = 3). (N, O) SCC-23 cells were co-transfected with the indicated constructs, and PFKP ubiquitination was examined under conditions of altered HSP90 expression (*n* = 3). Data are presented as mean ± SD unless otherwise indicated. Two-group comparisons were performed using two-tailed unpaired Student's *t*-test. ∗∗∗*P* < 0.001.

Since PLOD1 physically associates with PFKP, we next investigated whether PLOD1 influences PFKP protein stability. Treatment with CHX revealed that PLOD1 overexpression markedly prolonged the half-life of PFKP in both SCC-1 and SCC-23 cells, whereas PLOD1 depletion accelerated PFKP degradation (Fig. 7A–D). These findings suggest that PLOD1 stabilizes PFKP at the post-translational level. To determine the mechanism of PFKP degradation, we treated cells with the proteasome inhibitor MG132 or the lysosome inhibitor chloroquine. MG132 restored PFKP protein levels in PLOD1-deficient cells (Fig. 7E and Fig. S8C), whereas chloroquine had no significant effect (Fig. 7F and G, Fig. S8D and S8E). Immunoprecipitation of PFKP followed by immunoblotting with anti-ubiquitin antibodies revealed that PFKP ubiquitination was markedly increased in PLOD1-deficient cells and decreased upon PLOD1 overexpression (Supporting Information Fig. S9A–S9D). These results indicate that PLOD1 stabilizes PFKP by suppressing its ubiquitin-mediated proteasomal degradation.

### 3.7 PLOD1 enhances PFKP stability *via* HSP90-facilitated AKT phosphorylation at Ser386

Previous studies have shown that phosphorylation of PFKP enhances its stability and protects it from ubiquitin-mediated proteasomal degradation^23^, ^24^, ^25^. To test whether PFKP loss in PLOD1-deficient cells is linked to dephosphorylation, we first examined whether inhibiting phosphatase activity could restore PFKP levels. Treatment with a broad-spectrum phosphatase inhibitor cocktail (PIC) rescued PFKP protein expression in sgPLOD1 SCC-1 and SCC-23 cells, whereas the vehicle control had no effect (Fig. S9E and S9F). Phosphorylation of PFKP at serine 386 (S386) has been shown to play a critical role in maintaining its protein stability. To evaluate the functional relevance of this site, we generated a non-phosphorylatable S386A mutant and compared its stability with that of wild-type PFKP in PLOD1-overexpressing SCC-1 and SCC-23 cells. CHX chase assays demonstrated that the S386A mutant degraded more rapidly than the wild-type protein (Fig. 7H and I), indicating that S386 phosphorylation is required for PLOD1-mediated stabilization of PFKP.

Since S386 of PFKP has been reported as a target of AKT-mediated phosphorylation, we next investigated whether the PLOD1-induced increase in PFKP phosphorylation depends on AKT activity^26^. PLOD1 overexpression markedly enhanced the Ser/Thr phosphorylation of co-immunoprecipitated PFKP, and this effect was completely abolished by treatment with the AKT inhibitor MK-2206 (Fig. S9G and S9H).

HSP90 is a molecular chaperone that plays a critical role in maintaining AKT protein stability^27^^,^^28^. To explore whether PLOD1 intersects with this pathway, we performed GSEA of RNA-seq data, which revealed significant downregulation of heat shock protein binding signatures upon PLOD1 depletion (Supporting Information Fig. S10A). This observation prompted us to test whether PLOD1 physically associates with HSP90. Co-immunoprecipitation assays demonstrated that endogenous PLOD1 interacts with HSP90 in both SCC-1 and SCC-23 cells (Fig. S10B). Expectedly, HSP90 depletion markedly reduced the Ser/Thr phosphorylation of PFKP in both SCC-1 and SCC-23 cells (Fig. S10C and S10D). Given that HSP90 is essential for AKT stability and activity, we next examined whether PLOD1 modulates the HSP90–AKT interaction. Increasing PLOD1 expression enhanced the association between HSP90 and AKT, as shown by co-immunoprecipitation (Fig. 7J and Fig. S10E). Conversely, PLOD1 knockdown weakened this interaction (Fig. 7K, Fig. S10F), suggesting that PLOD1 facilitates the formation or stabilization of the HSP90–AKT complex. In both SCC-1 and SCC-23 cells, co-expression of HSP90 and PLOD1 led to a further reduction in PFKP ubiquitination compared to PLOD1 alone (Fig. 7L and M). Conversely, HSP90 knockdown markedly impaired the ability of PLOD1 to suppress PFKP ubiquitination (Fig. 7N and O). These findings indicate that HSP90 functionally cooperates with PLOD1 to restrain PFKP ubiquitination by supporting its phosphorylation-dependent stabilization.

To determine whether PFKP functions as a critical downstream effector of PLOD1, we re-expressed either PFKP-WT or PFKP-Mut in PLOD1-deficient SCC-1 and SCC-23 cells. While PFKP-WT substantially restored clonogenic growth, tumor sphere formation, and invasive capacity suppressed by PLOD1 loss, the S386A mutant was unable to rescue these phenotypes (Supporting Information Fig. S11A–S11D). In PLOD1-deficient SCC-1 and SCC-23 cells, reintroduction of PFKP-WT restored mesenchymal markers (N-cadherin, vimentin), suppressed E-cadherin, and reactivated stemness-associated proteins including SOX2, CD44, and CD133, whereas the S386A mutant had no discernible effect (Fig. S11E). In line with these observations, PFKP-WT—but not the S386A mutant—rescued lactate production (Fig. S11F and S11G). Importantly, tumor growth was markedly impaired upon PLOD1 loss, and re-expression of PFKP-WT substantially restored both tumor weight and volume, whereas the S386A mutant was unable to rescue tumor growth (Supporting Information Fig. S12A–S12C). These results collectively establish PFKP—specifically its phosphorylation at Ser386—as an essential mediator of PLOD1-driven metabolic reprogramming and malignant progression in HNSCC.

Although PFKP catalyzes a downstream glycolytic step in which fructose-6-phosphate (F6P) is phosphorylated to fructose-1,6-bisphosphate, we observed that PLOD1 depletion led not only to reduced PFKP stability but also to a marked decrease in glucose uptake and upstream intermediates such as F6P. This raised the possibility that PLOD1 regulates glycolytic flux through mechanisms beyond its effect on PFKP. Because glucose uptake is predominantly governed by AKT-mediated control of GLUT1 membrane trafficking, we next examined whether PLOD1 influences this process. PLOD1 depletion markedly diminished AKT signaling, prompting us to assess GLUT1 surface localization. Cell-surface biotinylation followed by immunoblotting revealed that loss of PLOD1 substantially decreased membrane-localized GLUT1 without altering its total protein abundance, whereas PLOD1 overexpression increased the plasma-membrane pool of GLUT1 (Supporting Information Fig. S13A–S13D). These findings indicate that PLOD1 regulates glucose entry by modulating AKT-dependent GLUT1 trafficking, thereby shaping the availability of upstream glycolytic intermediates such as F6P.

### 3.8 Metabolic-epigenetic coupling by PFKP mediates PLOD1-driven TGFBR2 activation in HNSCC

**Figure 8:**
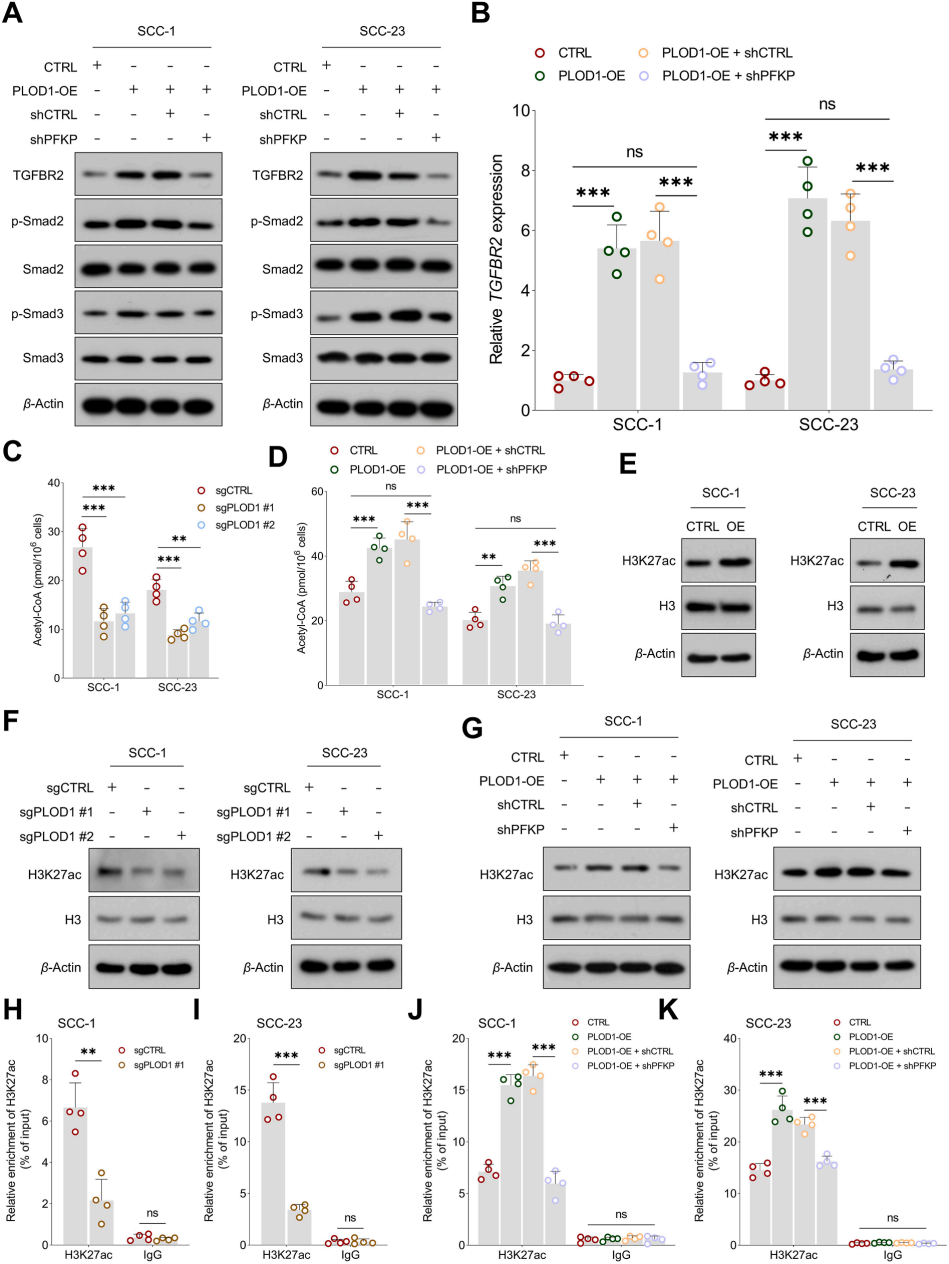
PLOD1 enhances TGFBR2 expression through PFKP-dependent acetyl-CoA production and histone acetylation. (A) Immunoblot analysis of TGFBR2, *p*-Smad2, total Smad2, *p*-Smad3, and total Smad3 in SCC-1 and SCC-23 cells transduced with the indicated constructs (*n* = 3). (B) Quantitative RT-PCR analysis of *TGFBR2* mRNA expression in SCC-1 and SCC-23 cells under the indicated conditions (*n* = 4). (C) Quantification of acetyl-CoA levels in SCC-1 and SCC-23 cells with PLOD1 knockout (*n* = 4). (D) Measurement of acetyl-CoA levels in SCC-1 and SCC-23 cells overexpressing PLOD1 with or without PFKP knockdown (*n* = 4). (E) Immunoblot analysis of H3K27ac levels in SCC-1 and SCC-23 cells with or without PLOD1 overexpression (*n* = 3). (F) H3K27ac levels were examined by immunoblotting in SCC-1 and SCC-23 cells following PLOD1 knockout (*n* = 3). (G) Immunoblot analysis of H3K27ac levels in SCC-1 and SCC-23 cells overexpressing PLOD1, with or without PFKP knockdown (*n* = 3). (H, I) ChIP-qPCR analysis of H3K27ac enrichment at the *TGFBR2* promoter in SCC-1 and SCC-23 cells with PLOD1 knockout. IgG was used as a negative control (*n* = 4). (J, K) ChIP-qPCR analysis of H3K27ac enrichment at the *TGFBR2* promoter in SCC-1 and SCC-23 cells overexpressing PLOD1, with or without PFKP knockdown (*n* = 4). Data are presented as mean ± SD unless otherwise indicated. Two-group comparisons were performed using two-tailed unpaired Student's *t*-test. For comparisons among multiple groups, one-way ANOVA with *post hoc* multiple-comparisons testing was used. ∗∗*P* < 0.01, ∗∗∗*P* < 0.001; ns, not significant.

We next investigated whether the glycolytic shift induced by PFKP contributes to PLOD1-mediated transcriptional activation of TGFBR2. In both SCC-1 and SCC-23 cells, PLOD1 overexpression markedly increased TGFBR2 protein levels and enhanced phosphorylation of its downstream effectors Smad2 and Smad3. These effects were abrogated upon PFKP knockdown (Fig. 8A). Consistently, qRT-PCR analysis revealed that the upregulation of *TGFBR2* mRNA by PLOD1 was also abolished in the absence of PFKP (Fig. 8B), indicating that PFKP is required for PLOD1-driven activation of the TGF-*β* receptor axis.

Given that glycolysis produces acetyl-CoA—a key metabolite that serves as a substrate for histone acetylation—we hypothesized that PFKP-dependent metabolic reprogramming may modulate TGFBR2 expression through epigenetic regulation^29^, ^30^, ^31^. Supporting this, PLOD1 knockout significantly reduced intracellular acetyl-CoA levels in both SCC-1 and SCC-23 cells, whereas PLOD1 overexpression elevated acetyl-CoA abundance in a manner dependent on PFKP (Fig. 8C and D). In parallel, global levels of H3K27 acetylation, a chromatin mark associated with transcriptional activation, were reduced following PLOD1 loss and elevated upon PLOD1 overexpression (Fig. 8E and F). Notably, the PLOD1-induced increase in H3K27ac was abolished by PFKP silencing (Fig. 8G), suggesting that glycolysis-driven acetyl-CoA production supports histone acetylation. To examine whether these epigenetic changes occur at the *TGFBR2* promoter, we performed ChIP-qPCR analysis of H3K27ac occupancy. PLOD1 depletion significantly reduced H3K27ac enrichment at the *TGFBR2* promoter in both cell lines (Fig. 8H and I). Conversely, PLOD1 overexpression enhanced promoter-associated H3K27ac, but this effect was fully dependent on PFKP (Fig. 8J and K). Together, these findings indicate that PFKP couples glycolytic flux to chromatin modification, enabling PLOD1 to transcriptionally activate TGFBR2 through an epigenetic mechanism.

Having established this link between metabolic flux and chromatin modification, we next asked whether acetyl-CoA availability functionally contributes to the phenotypes associated with PLOD1 loss. To test this, we supplemented sodium acetate in PLOD1-deficient cells. Acetate treatment partially restored global H3K27ac levels and H3K27ac enrichment at the *TGFBR2* promoter (Supporting Information Fig. S14A and S14B). Consistently, TGFBR2 expression and EMT/stemness markers—including E-cadherin, N-cadherin, vimentin and SOX2—were partially rescued (Fig. S14C and S14D). Functionally, acetate supplementation also partially reversed the impaired spheroid formation and clonogenic capacity observed in PLOD1-deficient cells exposed to cisplatin (Fig. S14E–S14H). These results demonstrate that reduced acetyl-CoA contributes to the EMT, stemness and cisplatin-resistant phenotypes downstream of PLOD1 loss.

We next examined whether decreasing acetyl-CoA availability is sufficient to counteract the phenotypes induced by PLOD1 overexpression. To this end, we silenced ATP citrate lyase (ACLY), a major enzyme generating acetyl-CoA. ACLY knockdown lowered acetyl-CoA levels and reduced both global H3K27ac and H3K27ac enrichment at the *TGFBR2* promoter in PLOD1-overexpressing cells (Supporting Information Fig. S15A–S15D). This metabolic attenuation was accompanied by decreased TGFBR2 expression and a shift toward a more epithelial and less stem-like marker profile, including changes in E-cadherin, N-cadherin, vimentin and SOX2 (Fig. S15E and S15F). Functionally, ACLY depletion also weakened the enhanced spheroid formation and clonogenic growth driven by PLOD1 overexpression under cisplatin treatment (Fig. S15G–S15J). Together, these findings demonstrate that acetyl-CoA availability is a critical determinant of the EMT, stemness and cisplatin-resistant states induced by PLOD1 overexpression and that targeting acetyl-CoA synthesis can mitigate these downstream effects.

### 3.9 PLOD1 ablation enhances cisplatin sensitivity and synergistically limits HNSCC tumor growth and metastasis *in vivo*

**Figure 9:**
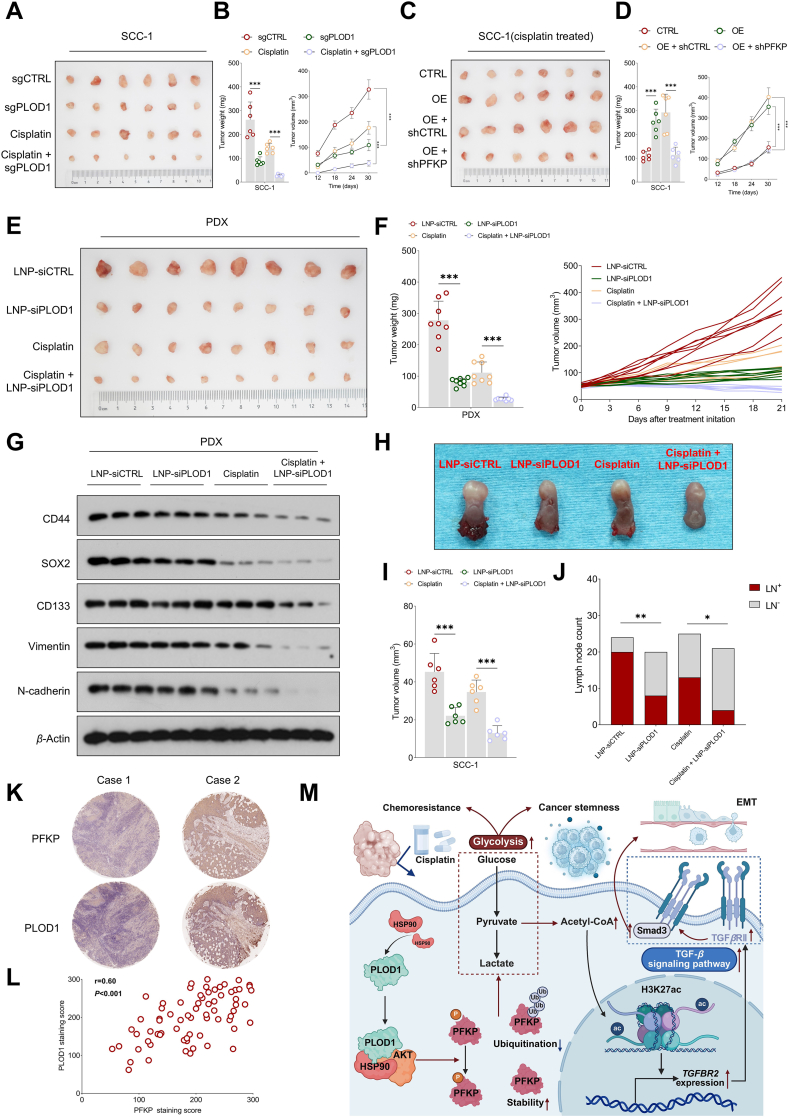
*In vivo* modeling reveals the role of the PLOD1–PFKP axis in driving cisplatin resistance in HNSCC. (A, B) Subcutaneous xenograft models were established using SCC-1 cells with or without PLOD1 knockout, followed by treatment with cisplatin or vehicle, to evaluate the combined effects of PLOD1 deficiency and cisplatin on tumor progression (*n* = 6). (C, D) SCC-1 cells overexpressing PLOD1, with or without PFKP knockdown, were implanted subcutaneously into immunodeficient mice. Mice were treated with cisplatin to assess whether PFKP is required for PLOD1-mediated tumor growth under chemotherapeutic stress (*n* = 6). (E, F) PDX tumors were treated with LNP-formulated siRNAs targeting PLOD1 (LNP-siPLOD1) or a non-targeting control (LNP-siCTRL), in combination with or without cisplatin, to evaluate the combined therapeutic effect of PLOD1 depletion and cisplatin treatment (*n* = 8). (G) Immunoblot analysis of CD44, SOX2, CD133, Vimentin, N-cadherin in PDX tumors from the indicated treatment groups (*n* = 3). (H, I) Orthotopic xenograft models were established by injecting HNSCC cells into the tongue of immunodeficient mice, followed by treatment with LNP-siPLOD1 or LNP-siCTRL, with or without cisplatin (*n* = 6). (J) Incidence of cervical lymph node metastasis in orthotopic tumor-bearing mice following the indicated treatments. (K, L) Immunohistochemical analysis and correlation of PFKP and PLOD1 expression in an in-house primary HNSCC cohort. (M) Schematic model illustrating the role of PLOD1 in promoting cancer stemness and chemoresistance through metabolic and epigenetic regulation. Data are presented as mean ± SD unless otherwise indicated. For comparisons among multiple groups, one-way ANOVA with *post hoc* multiple-comparisons testing was used. ∗*P* < 0.05, ∗∗*P* < 0.01, ∗∗∗*P* < 0.001.

To evaluate the therapeutic potential of targeting PLOD1, we first assessed whether PLOD1 ablation could enhance the efficacy of cisplatin *in vivo*. In SCC-1 xenograft models, cisplatin monotherapy led to a modest reduction in tumor growth, whereas PLOD1 knockout alone exerted a more substantial inhibitory effect. Notably, the combination of PLOD1 deletion and cisplatin treatment resulted in a synergistic suppression of tumor volume and weight (Fig. 9A and B). In parallel, knockdown of PFKP abrogated the tumor-promoting effect of PLOD1 overexpression under cisplatin treatment, reinforcing the requirement of PFKP for PLOD1-driven chemoresistance (Fig. 9C and D).

To investigate translational applicability, we employed a lipid nanoparticle (LNP)-mediated siRNA delivery platform to silence PLOD1 in a PDX model. LNP-encapsulated PLOD1 siRNA (LNP-siPLOD1) significantly inhibited tumor growth relative to LNP-siCTRL. Moreover, combined treatment with LNP-siPLOD1 and cisplatin yielded a more pronounced therapeutic benefit than either agent alone (Fig. 9E and F). Western blot analysis of harvested tumors showed that PLOD1 silencing downregulated the expression of stemness-associated markers (SOX2, CD44, CD133) and mesenchymal proteins (vimentin, N-cadherin), indicating reversal of stem-like and EMT phenotypes (Fig. 9G). We next examined whether PLOD1 targeting is effective in a cisplatin-resistant context. As shown in Supporting Information Fig. S16A–S16C, tumors in this model exhibited comparable growth under cisplatin and vehicle treatment, confirming minimal intrinsic sensitivity to cisplatin. Within this resistant background, LNP-mediated delivery of siPLOD1 in combination with cisplatin produced a marked suppression of tumor growth, indicating that PLOD1 depletion can restore cisplatin responsiveness in resistant tumors. To further assess the impact of PLOD1 inhibition on metastatic progression, we employed an orthotopic SCC-1 tumor model. Both LNP-siPLOD1 and cisplatin modestly suppressed primary tumor burden, while their combination produced a synergistic reduction in tumor size (Fig. 9H and I). Analysis of lymph nodes revealed that dual treatment significantly reduced the number and extent of nodal metastases (Fig. 9J), underscoring the therapeutic potential of PLOD1 targeting in controlling both tumor growth and dissemination.

To explore the clinical relevance of the PLOD1–PFKP axis, we analyzed a tissue microarray derived from an in-house primary HNSCC cohort. Representative staining revealed a concordant increase in PLOD1 and PFKP protein levels across tumor specimens (Fig. 9K). Quantitative analysis demonstrated a strong positive correlation between PLOD1 and PFKP expression (*r* = 0.60, *P* < 0.001; Fig. 9L).

## 4 Discussion

Cisplatin resistance remains a major obstacle to effective therapy in HNSCC, yet the metabolic mechanisms sustaining this phenotype remain incompletely understood^28^^,^^32^^,^^33^. Here, we identify the PLOD1–PFKP–glycolysis axis as a critical driver of resistance, linking post-translational protein stabilization to sustained metabolic reprogramming (Fig. 9M). Mechanistically, PLOD1 enhances the stability of PFKP—a rate-limiting glycolytic enzyme—thereby maintaining elevated glycolytic flux independently of transcriptional regulation. This persistent metabolic state provides a continuous supply of ATP and anabolic precursors, supporting cell survival under cisplatin-induced stress. In parallel, enhanced glycolysis facilitates redox homeostasis and promotes the emergence of phenotypically plastic, therapy-tolerant cell populations. In this context, the PLOD1–PFKP axis functions as a central metabolic-regulatory hub that reinforces drug resistance through coordinated bioenergetic and adaptive mechanisms.

In designing our analytical workflow, we did not rely solely on differential gene expression between cisplatin-sensitive and cisplatin-resistant PDX tumors. Although such a comparison appears intuitive, the limited number of biologically independent PDX samples substantially constrains statistical power and increases susceptibility to stochastic variation, making it challenging to identify robust resistance-associated genes. To ensure that the candidate regulators we prioritized—including PLOD1—reflected biologically meaningful and clinically relevant alterations, we first leveraged the TCGA HNSCC cohort to identify genes that are consistently upregulated in primary tumors and associated with poor prognosis. This large-scale dataset provides the statistical rigor and clinical breadth necessary to nominate reliable tumor-associated drivers. We subsequently examined whether these TCGA-derived genes, including PLOD1, also stratify cisplatin response in our PDX models. This two-tiered strategy substantially reduces noise inherent to small-sample PDX comparisons and ensures that the targets evaluated *in vivo* are anchored in human tumor biology rather than sample-to-sample variability. By integrating patient-derived datasets with functional validation in PDX models, this framework strengthens the rationale for selecting PLOD1 as a mechanistically relevant and translationally actionable determinant of cisplatin resistance.

Mechanistically, our study uncovers a non-canonical function of PLOD1 in regulating metabolic adaptation through post-translational protein stabilization. Traditionally recognized for its role in collagen lysyl hydroxylation within the endoplasmic reticulum, PLOD1 here is shown to exert extramural influence by directly interacting with cytoplasmic signaling complexes^34^. Specifically, PLOD1 facilitates the assembly and stabilization of the HSP90–AKT complex, a stress-responsive chaperone-kinase module known to maintain signaling homeostasis under adverse conditions. HSP90 acts as a central molecular chaperone that not only stabilizes a broad array of client proteins—including kinases, transcription factors, and metabolic enzymes—but also protects them from proteasomal degradation by shielding them from E3 ligase access. In this context, HSP90 serves as a structural scaffold that sustains AKT activity and ensures the fidelity of signal transduction required for cellular adaptation during chemotherapeutic stress.

AKT, in turn, is a key serine/threonine kinase that modulates diverse cellular functions including metabolism, survival, and protein turnover^35^, ^36^, ^37^. We demonstrate that PLOD1 promotes AKT-mediated phosphorylation of PFKP at serine 386, a post-translational modification previously associated with enhanced protein stability and glycolytic activation. Phosphorylation at this site appears to block the recruitment of ubiquitin E3 ligases, thereby preventing PFKP from undergoing proteasomal degradation. This finding integrates two regulatory layers: chaperone-mediated complex stabilization and kinase-driven protection from ubiquitination. Importantly, disruption of either HSP90 or AKT signaling impairs PLOD1-mediated PFKP stabilization, underscoring the hierarchical interdependence of these components in maintaining glycolytic fidelity. This mechanism highlights a broader paradigm in which metabolic enzymes are not merely regulated at the transcriptional level but are also subject to dynamic post-translational control involving protein–protein interactions and modification networks. It further positions PLOD1 as a moonlighting protein with roles that extend beyond its classical enzymatic function. Such non-canonical regulatory behaviors are increasingly recognized as critical determinants of drug resistance and metabolic plasticity in cancer. The PLOD1–HSP90–AKT–PFKP signaling axis thus represents a previously unappreciated convergence point of chaperone biology, kinase signaling, and metabolic enzyme regulation, offering novel opportunities for therapeutic intervention in cisplatin-resistant HNSCC.

Importantly, our results reveal that the glycolytic program initiated by the PLOD1–PFKP axis extends into the epigenetic regulation of gene expression through metabolite-driven histone modification. Among these, H3K27ac is a canonical mark of active enhancers and promoters, often associated with increased chromatin accessibility and transcriptional initiation^38^, ^39^, ^40^. This epigenetic modification, catalyzed by histone acetyltransferases in an acetyl-CoA-dependent manner, plays a pivotal role in establishing cell-type-specific gene expression programs and enabling dynamic transcriptional responses to environmental cues^41^^,^^42^. In this context, we observe a marked elevation of H3K27ac at the promoter region of *TGFBR2*, a key mediator of the TGF-*β* signaling cascade. This chromatin change is tightly associated with transcriptional activation, suggesting that glycolysis-driven acetyl-CoA accumulation directly facilitates the epigenetic upregulation of TGFBR2. Such metabolic–epigenetic coupling provides a mechanistic link between intracellular metabolic state and the activation of lineage-instructive transcriptional circuits that underlie tumor cell plasticity. TGF-*β* signaling is a well-established driver of phenotypic diversification and cellular motility in epithelial malignancies, and its sustained activation promotes the acquisition of mesenchymal-like features associated with therapeutic tolerance^43^. By enhancing TGFBR2 expression *via* H3K27 acetylation, the glycolytic program downstream of PFKP stabilization extends beyond metabolic adaptation to enforce an epigenetically encoded transcriptional state that supports drug resistance. Notably, this regulatory axis operates independently of canonical oncogenic mutations, instead relying on metabolite availability to orchestrate chromatin remodeling and gene activation—highlighting a previously underappreciated layer of regulation that links metabolic flux to therapy-refractory cellular phenotypes.

To evaluate the translational relevance of targeting the PLOD1–PFKP axis, we employed both PDX models and an immunocompetent orthotopic tongue cancer model. In both systems, treatment with LNP-formulated siRNAs against PLOD1 in combination with cisplatin elicited a synergistic antitumor response, significantly reducing tumor burden. Notably, in the orthotopic model, this combinatorial strategy also markedly suppressed lymph node metastasis, underscoring its potential to restrict locoregional disease progression. These findings highlight the feasibility and therapeutic promise of RNA-based PLOD1 inhibition in overcoming cisplatin resistance. Nevertheless, to enhance clinical applicability and broaden therapeutic reach, the development of small-molecule inhibitors that target PLOD1 with high specificity and favorable pharmacological properties may represent a complementary or alternative approach. Such agents could disrupt the metabolic–epigenetic circuitry maintained by the PLOD1–PFKP axis and synergize with existing chemotherapies to improve patient outcomes.

## 5 Conclusions

In conclusion, this study identifies the PLOD1–PFKP–glycolysis axis as a central mediator of cisplatin resistance in HNSCC, operating through both metabolic adaptation and epigenetic remodeling. By stabilizing PFKP and sustaining glycolytic flux, PLOD1 promotes a therapy-tolerant cellular phenotype that is further reinforced by histone acetylation-dependent activation of TGFBR2. These findings provide mechanistic insights into the metabolic basis of drug resistance and support further exploration of PLOD1 as a therapeutic target in treatment-refractory HNSCC, potentially enabling new combination strategies to overcome chemoresistance in clinical settings.

## Author contributions

Xinyuan Zhao: conceptualization, methodology, experiments, data curation, supervision, writing–original draft and writing–review & editing. Xin Liao: experiments, data curation, methodology. Yunfan Lin: experiments, data curation, methodology, writing–original draft. Xu Chen: experiments and data curation. Pei Lin: experiments. Ye Lu: experiments and data curation. Jiarong Zheng: experiments and data curation. Meiyan Zou: experiments. Bing Guo: conceptualization, supervision and writing–review & editing. Li Cui: conceptualization, methodology, experiments, data curation, supervision, writing–original draft and writing–review & editing.

## Conflicts of interest

The authors declare no conflicts of interest.

## Appendix A Supporting information

The following is the Supporting Information to this article.

## References

[1] Sung H., Ferlay J., Siegel R.L., Laversanne M., Soerjomataram I., Jemal A. (2021). Global cancer statistics 2020: GLOBOCAN estimates of incidence and mortality worldwide for 36 cancers in 185 countries. CA Cancer J Clin.

[2] Siegel R.L., Giaquinto A.N., Jemal A. (2024). Cancer statistics, 2024. CA Cancer J Clin.

[3] O'Leary B., Skinner H., Schoenfeld J.D., Licitra L., Le Tourneau C., Esdar C. (2024). Evasion of apoptosis and treatment resistance in squamous cell carcinoma of the head and neck. Cancer Treat Rev.

[4] Liang D., Yu C., Ma Z., Yang X., Li Z., Dong X. (2022). Identification of anthelmintic parbendazole as a therapeutic molecule for HNSCC through connectivity map-based drug repositioning. Acta Pharm Sin B.

[5] Rohde M., Nielsen A.L., Pareek M., Johansen J., Sørensen J.A., Diaz A. (2019). PET/CT *versus* standard imaging for prediction of survival in patients with recurrent head and neck squamous cell carcinoma. J Nucl Med.

[6] Zhao X., Guo B., Sun W., Yu J., Cui L. (2023). Targeting squalene epoxidase confers metabolic vulnerability and overcomes chemoresistance in HNSCC. Adv Sci (Weinh).

[7] Li G., Che X., Wang S., Liu D., Xie D., Jiang B. (2025). The role of cisplatin in modulating the tumor immune microenvironment and its combination therapy strategies: a new approach to enhance anti-tumor efficacy. Ann Med.

[8] Cui L., Lu Y., Zheng J., Guo B., Zhao X. (2023). ACTN1 promotes HNSCC tumorigenesis and cisplatin resistance by enhancing MYH9-dependent degradation of GSK-3*β* and integrin *β*1-mediated phosphorylation of FAK. J Exp Clin Cancer Res.

[9] Lengrand J., Pastushenko I., Vanuytven S., Song Y., Venet D., Sarate R.M. (2023). Pharmacological targeting of netrin-1 inhibits EMT in cancer. Nature.

[10] Liu S., Zhang X., Wang W., Li X., Sun X., Zhao Y. (2024). Metabolic reprogramming and therapeutic resistance in primary and metastatic breast cancer. Mol Cancer.

[11] Morandi A., Indraccolo S. (2017). Linking metabolic reprogramming to therapy resistance in cancer. Biochim Biophys Acta Rev Cancer.

[12] Hanahan D. (2022). Hallmarks of cancer: new dimensions. Cancer Discov.

[13] Zhao X., Mai Z., Liu L., Lu Y., Cui L., Yu J. (2024). Hypoxia-driven TNS4 fosters HNSCC tumorigenesis by stabilizing integrin *α5β*1 complex and triggering FAK-mediated Akt and TGF*β* signaling pathways. Int J Biol Sci.

[14] Lazure F., Gomes A.P. (2025). Cancer progression through the lens of age-induced metabolic reprogramming. Nat Rev Cancer.

[15] Cui L., Zhao X., Jin Z., Wang H., Yang S.F., Hu S. (2021). Melatonin modulates metabolic remodeling in HNSCC by suppressing MTHFD1L–formate axis. J Pineal Res.

[16] Ganapathy-Kanniappan S., Geschwind J.F. (2013). Tumor glycolysis as a target for cancer therapy: progress and prospects. Mol Cancer.

[17] Huang P.C., Chang C.W., Lin Y.C., Chen C.Y., Chen T.Y., Chuang L.T. (2023). Pyruvate kinase differentially alters metabolic signatures during head and neck carcinogenesis. Int J Mol Sci.

[18] Loh J.J., Ma S. (2024). Hallmarks of cancer stemness. Cell Stem Cell.

[19] Zhao X., Shu D., Sun W., Si S., Ran W., Guo B. (2022). PLEK2 promotes cancer stemness and tumorigenesis of head and neck squamous cell carcinoma *via* the c-Myc-mediated positive feedback loop. Cancer Commun.

[20] Agudo J., Miao Y. (2025). Stemness in solid malignancies: coping with immune attack. Nat Rev Cancer.

[21] Zhang Y., Wu Y., Su X. (2021). PLOD1 promotes cell growth and aerobic glycolysis by regulating the SOX9/PI3K/Akt/mTOR signaling pathway in gastric cancer. Front Biosci (Landmark Ed).

[22] Zhang C., Zhou Y., Hu M., Pan Y., Chen X., Sun Q. (2025). PLOD1 promotes the malignancy of hepatocellular carcinoma by facilitating the NF-kappaB/IL-6/STAT3-dependent TCA cycle. JHEP Rep.

[23] Jeon S.M., Lim J.S., Park S.H., Lee J.H. (2021). Wnt signaling promotes tumor development in part through phosphofructokinase 1 platelet isoform upregulation. Oncol Rep.

[24] Lee J.H., Liu R., Li J., Wang Y., Tan L., Li X.J. (2018). EGFR-phosphorylated platelet isoform of phosphofructokinase 1 promotes PI3K activation. Mol Cell.

[25] Lim J.S., Shi Y., Park S.H., Jeon S.M., Zhang C., Park Y.Y. (2022). Mutual regulation between phosphofructokinase 1 platelet isoform and VEGF promotes glioblastoma tumor growth. Cell Death Dis.

[26] Lee J.H., Liu R., Li J., Zhang C., Wang Y., Cai Q. (2017). Stabilization of phosphofructokinase 1 platelet isoform by AKT promotes tumorigenesis. Nat Commun.

[27] Sato S., Fujita N., Tsuruo T. (2000). Modulation of Akt kinase activity by binding to Hsp90. Proc Natl Acad Sci U S A.

[28] Basso A.D., Solit D.B., Chiosis G., Giri B., Tsichlis P., Rosen N. (2002). Akt forms an intracellular complex with heat shock protein 90 (Hsp90) and Cdc37 and is destabilized by inhibitors of Hsp90 function. J Biol Chem.

[29] Liao M., Yao D., Wu L., Luo C., Wang Z., Zhang J. (2024). Targeting the Warburg effect: a revisited perspective from molecular mechanisms to traditional and innovative therapeutic strategies in cancer. Acta Pharm Sin B.

[30] Paul S., Ghosh S., Kumar S. (2022). Tumor glycolysis, an essential sweet tooth of tumor cells. Semin Cancer Biol.

[31] Li X., Yang Y., Zhang B., Lin X., Fu X., An Y. (2022). Lactate metabolism in human health and disease. Signal Transduct Targeted Ther.

[32] Ruffin A.T., Li H., Vujanovic L., Zandberg D.P., Ferris R.L., Bruno T.C. (2023). Improving head and neck cancer therapies by immunomodulation of the tumour microenvironment. Nat Rev Cancer.

[33] Khera N., Rajkumar A.S., Abdulkader M.A.K., Liu Z., Ma H., Waseem A. (2023). Identification of multidrug chemoresistant genes in head and neck squamous cell carcinoma cells. Mol Cancer.

[34] Qi Y., Xu R. (2018). Roles of PLODs in collagen synthesis and cancer progression. Front Cell Dev Biol.

[35] Dibble C.C., Barritt S.A., Perry G.E., Lien E.C., Geck R.C., DuBois-Coyne S.E. (2022). PI3K drives the *de novo* synthesis of coenzyme A from vitamin B5. Nature.

[36] Chen T., Xie S., Cheng J., Zhao Q., Wu H., Jiang P. (2024). AKT1 phosphorylation of cytoplasmic ME2 induces a metabolic switch to glycolysis for tumorigenesis. Nat Commun.

[37] Hoxhaj G., Manning B.D. (2020). The PI3K–AKT network at the interface of oncogenic signalling and cancer metabolism. Nat Rev Cancer.

[38] Creyghton M.P., Cheng A.W., Welstead G.G., Kooistra T., Carey B.W., Steine E.J. (2010). Histone H3K27ac separates active from poised enhancers and predicts developmental state. Proc Natl Acad Sci U S A.

[39] Zheng Q., Li X., Li F., Xu Z., Cai Y., Li C. (2025). Temporal-spatially metabolic reprogramming rewires the landscape of H3K27 acetylation to enable the initiation of liver regeneration. Sci Bull (Beijing).

[40] Hnisz D., Abraham B.J., Lee T.I., Lau A., Saint-André V., Sigova A.A. (2013). Super-enhancers in the control of cell identity and disease. Cell.

[41] Huang X., Zhang J., Cun Y., Ye M., Ren Z., Guo W. (2025). Spatial control of m^6^A deposition on enhancer and promoter RNAs through co-acetylation of METTL3 and H3K27 on chromatin. Mol Cell.

[42] Ge J., Meng Y., Guo J., Chen P., Wang J., Shi L. (2024). Human papillomavirus-encoded circular RNA circE7 promotes immune evasion in head and neck squamous cell carcinoma. Nat Commun.

[43] Zhang J., Zhang Z., Huang Z., Li M., Yang F., Wu Z. (2023). Isotoosendanin exerts inhibition on triple-negative breast cancer through abrogating TGF-beta-induced epithelial–mesenchymal transition *via* directly targeting TGFbetaR1. Acta Pharm Sin B.

