# Supplementary material for: Metabolic-epigenetic reprogramming via the PLOD1–PFKP axis drives cisplatin resistance in HNSCC
Source: Acta Pharm Sin B. 2026 Apr 3;16(8):5108–30. doi: 10.1016/j.apsb.2026.03.053 (PMC13464578; doi:10.1016/j.apsb.2026.03.053)
Supplement: Supplementary file 1 [file mmc1.docx]

**Supporting Information for**

**Original article**

**Metabolic-epigenetic reprogramming *via* the PLOD1–PFKP axis drives cisplatin resistance in HNSCC**

**Xinyuan Zhao^a^, Xin Liao^b^, Yunfan Lin^a^, Xu Chen^a^, Pei Lin^a^, Ye Lu^a^, Jiarong Zheng^b^, Meiyan Zou^a^, Bing Guo^b^, Li Cui^a,c,^**^*^

^a^*Stomatological Hospital, School of Stomatology, Southern Medical University, Guangzhou 510280, China*

^b^*Department of Dentistry, The First Affiliated Hospital, Sun Yat-sen University, Guangzhou 510080, China*

^c^*School of Dentistry, University of California, Los Angeles, CA 90095, USA*

Received 30 September 2025; received in revised form 9 December 2025; accepted 13 January 2026

^*^Corresponding author.

E-mail address: [licui@smu.edu.cn](mailto:licui@smu.edu.cn) (Li Cui).

**Figure S1** Upregulation of PLOD1 in cisplatin-resistant tumors in the 4NQO-induced orthotopic HNSCC model. (A) Schematic representation of the 4NQO-induced oral carcinogenesis model. Mice were administered 4NQO in drinking water from weeks 0 to 16, followed by normal water until week 26. Mice bearing tongue tumors of comparable size were treated with cisplatin and subsequently classified as cisplatin-sensitive or cisplatin-resistant based on treatment response. (B) Immunoblot analysis of PLOD1 expression in tongue tumors from cisplatin-sensitive and cisplatin- resistant mice (*n*=12). (C) Immunoblot analysis of PLOD1 in normal human keratinocytes (NHOK and NHEK) and a panel of HNSCC cell lines with differing aggressiveness (*n*=3). Data are presented as mean ± SD unless otherwise indicated. Two-group comparisons were performed using two-tailed unpaired Student’s *t*-test. ****P* < 0.001.

**Figure S2** PLOD1 expression is elevated in HNSCC and predicts poor prognosis across independent patient cohorts. (A–C) Analysis of PLOD1 expression in paired tumor and adjacent non-tumor tissues from TCGA HNSCC and GSE127165. (D–I) Comparison of PLOD1 expression between normal and tumor tissues in GSE6631, GSE37991, GSE58911, GSE25099, GSE34105, and GSE143224. (J) PLOD1 expression in normal tissue, surgical margins, and tumor samples from patients with HNSCC in the GSE31056 dataset. (K) PLOD1 expression in normal mucosa, dysplastic lesions, and tumor tissues in the GSE30784 dataset. (L–N) Kaplan–Meier survival analysis showing overall survival in patients with high versus low PLOD1 expression from TCGA HNSCC, GSE65858, and GSE41613. *P* values were calculated using the log-rank test. ***P* < 0.001, ****P* < 0.001, *****P* < 0.001.

**Figure S3** Prognostic relevance of PLOD1 expression across multiple cancer types in TCGA. (A–P) Kaplan–Meier survival analyses of overall survival in patients with high versus low PLOD1 expression across 16 TCGA cancer types: CESC, CHOL, ESCA, GBM, KIRC, KIRP, LAML, LGG, LIHC, LUAD MESO, OV, PAAD, SARC, STAD, and UCS. *P* values were calculated using the log-rank test.

**Figure S4** PLOD1 promotes EMT independently of TGF-*β*1 production in HNSCC. (A, B) Immunoblot analysis of ALDH1A1 in SCC-1 and SCC-23 cells after PLOD1 knockdown or overexpression (*n*=3). (C, D) Spearman correlation analysis between *PLOD1* expression and EMT-associated genes in the TCGA HNSCC cohort. (E–G) Immunofluorescence staining of E-cadherin, N-cadherin, and Vimentin in SCC-23 cells with or without PLOD1 knockout (*n*=3). (H–K) Quantification of active and total TGF-*β*1 by ELISA in SCC-1 and SCC-23 cells following PLOD1 knockout or overexpression (*n*=6). Data are presented as mean ± SD unless otherwise indicated. Two-group comparisons were performed using two-tailed unpaired Student’s *t*-test. For comparisons among multiple groups, one-way ANOVA with *post hoc* multiple-comparisons testing was used. ns, not significant.

**Figure S5** PLOD1 promotes TGF-*β* signaling *via* upregulation of TGFBR2. (A) Quantitative RT-PCR analysis of *TGFBR1*, *TGFBR2*, *TGFBR3*, *PAI-1*, *MMP2*, *CTGF*, and *SNAI2* expression in SCC-23 cells with or without PLOD1 knockout (*n*=4). (B, C) Quantitative RT-PCR analysis of *TGFBR1*, *TGFBR2*, *TGFBR3*, *PAI-1*, *MMP2*, *CTGF*, and *SNAI2* expression in SCC-1 and SCC-23 cells with or without PLOD1 overexpression (*n*=4). (D) Immunoblot analysis of TGF-*β* receptors and Smad2/3 signaling in SCC-1 and SCC-23 cells with or without PLOD1 overexpression (*n*=3). (E, F) Colony formation assays of SCC-1 and SCC-23 cells overexpressing PLOD1, treated with LY2109761 (10 μmol/L) or DMSO, in the presence of cisplatin (*n*=4). (G, H) Immunoblot analysis of SOX2, CD44, CD133, E-cadherin, N-cadherin, Vimentin in SCC-1 and SCC-23 cells overexpressing PLOD1, following treatment with LY2109761 (10 μmol/L) or DMSO (*n*=3). Data are presented as mean ± SD unless otherwise indicated. Two-group comparisons were performed using two-tailed unpaired Student’s *t*-test. For comparisons among multiple groups, one-way ANOVA with *post hoc* multiple-comparisons testing was used. ***P* < 0.01, ****P* < 0.001; ns, not significant.

**Figure S6** PLOD1 regulates glycolytic metabolism in HNSCC cells. (A, B) Glucose uptake and lactate secretion in SCC-1 cells following PLOD1 depletion (*n*=4). (C, D) Relative glucose uptake and lactate production in SCC-1 cells with or without PLOD1 overexpression (*n*=4). (E, F) Quantification of glucose uptake and lactate production in SCC-23 cells following PLOD1 knockout (*n*=4). (G, H) Quantification of glucose uptake and lactate production in SCC-23 cells with or without enforced PLOD1 expression (*n*=4). (I, J) OCR analysis and quantification of basal and maximal respiration rates in SCC- 23 cells with PLOD1 depletion (*n*=3). (K, L) ECAR analysis and quantification of glycolysis rate and glycolysis capacity in SCC-23 cells with PLOD1 depletion (*n*=3). (M, N) OCR analysis and quantification of basal and maximal respiration in SCC-23 cells overexpressing PLOD1 (*n*=3). (O, P) ECAR analysis and quantification of glycolysis rate and glycolysis capacity in SCC-23 cells with or without PLOD1 overexpression (*n*=3). Data are presented as mean ± SD unless otherwise indicated. Two-group comparisons were performed using two-tailed unpaired Student’s *t*-test. For comparisons among multiple groups, one-way ANOVA with *post hoc* multiple-comparisons testing was used. **P* < 0.05, ***P* < 0.01, ****P* < 0.001.

**Figure S7** Functional dependency of PFKP in mediating PLOD1-induced oncogenic phenotypes and metabolic activity in HNSCC cells. (A–C) Assessment of colony formation, invasion, and tumorsphere formation in SCC-1 and SCC-23 cells overexpressing PLOD1 with or without PFKP knockdown (*n*=4). (D) Immunoblot analysis of E-cadherin, N-cadherin, vimentin, SOX2, CD44, CD133 in SCC-1 and SCC-23 cells transduced with indicated constructs (*n*=3). (E, F) OCR and ECAR analyses in SCC-1 cells overexpressing PLOD1 with or without PFKP knockdown to assess mitochondrial respiration and glycolytic flux (*n*=3). (G, H) OCR and ECAR analyses in SCC-23 cells overexpressing PLOD1, with or without concurrent PFKP knockdown (*n*=3). Data are presented as mean ± SD unless otherwise indicated. For comparisons among multiple groups, one-way ANOVA with *post hoc* multiple-comparisons testing was used. ****P* < 0.001; ns, not significant.

**Figure S8** PLOD1 interacts with PFKP and regulates its protein stability *via* the proteasome pathway. (A, B) Co-IP analysis of the interaction between exogenous PLOD1 and PFKP in SCC-23 cells is shown. The interaction between Flag-tagged PLOD1 and HA-tagged PFKP was immunoprecipitated using an anti-Flag antibody, and the reverse interaction between Flag-tagged PLOD1 and HA-tagged PFKP was immunoprecipitated using an anti-HA antibody (*n*=3). (C) Effect of MG132 treatment on PFKP levels in SCC-23 cells with PLOD1 depletion (*n*=3). (D, E) Assessment of changes in PFKP protein levels in SCC-23 cells with PLOD1 overexpression or depletion following chloroquine treatment (*n*=3).

**Figure S9** PLOD1 regulates PFKP ubiquitination and stability through phosphorylation-dependent mechanisms. (A–D) PFKP ubiquitination levels in SCC-1 and SCC-23 cells with PLOD1 knockout or overexpression, as determined by immunoprecipitation of PFKP followed by immunoblotting for ubiquitin (*n*=3). (E, F) Immunoblot analysis of PFKP protein levels in SCC-1 and SCC-23 cells with PLOD1 depletion, following treatment with DMSO or phosphatase inhibitor cocktail (PIC) (*n*=3). (G, H) Immunoprecipitation of endogenous PFKP followed by immunoblotting with a phospho-serine/threonine antibody to assess PFKP phosphorylation levels in SCC-1 and SCC-23 cells overexpressing Flag-PLOD1, with or without MK-2206 (*n*=3).

 **Figure S10** PLOD1 interacts with HSP90 and promotes HSP90-dependent phosphorylation of PFKP. (A) GSEA demonstrating enrichment of the HEAT_SHOCK_PROTEIN_BINDING gene set in SCC-1 cells transduced with control sgRNA compared to PLOD1 knockout. (B) Co-IP analysis showing reciprocal interaction between endogenous PLOD1 and HSP90 in SCC-1 and SCC-23 cells (*n*=3). (C, D) Immunoprecipitation of endogenous PFKP followed by immunoblotting with anti-phospho-serine/threonine antibody in SCC-1 and SCC-23cells transfected with siRNAs targeting HSP90. PFKP phosphorylation and associated proteins were assessed by immunoblotting (*n*=3). (E) Co-immunoprecipitation was performed in SCC-23 cells using an anti-HSP90 antibody to evaluate the interaction between HSP90 and AKT in the context of increasing PLOD1 expression levels (*n*=3). (F) SCC-23 cells transduced with control or PLOD1-targeting sgRNAs were subjected to co-immunoprecipitation with an anti-HSP90 antibody to determine the effect of PLOD1 depletion on the HSP90–AKT interaction (*n*=3).

**Figure S11** Phosphorylation of PFKP is required to restore stemness, invasion, and glycolytic activity in PLOD1-deficient HNSCC cells. (A) Immunoblot analysis of PFKP expression in SCC-1 and SCC-23 cells with PLOD1 knockout, reconstituted with PFKP-WT or PFKP-mutant (*n*=3). (B–D) Evaluation of colony formation, tumorsphere formation, and invasive capacity in SCC-1 and SCC-23 cells with PLOD1 knockout, reconstituted with control vector, PFKP-WT, or PFKP-mutant (*n*=4). (E) Immunoblot analysis of E-cadherin, N-cadherin, vimentin, SOX2, CD44, CD133 in SCC-1 and SCC-23 cells with indicated treatments (*n*=3). (F, G) Quantification of lactate production in SCC-1 and SCC-23 cells following PLOD1 knockout and reconstitution with control vector, PFKP-WT, or PFKP-mutant (*n*=4). Data are presented as mean ± SD unless otherwise indicated. For comparisons among multiple groups, one-way ANOVA with *post hoc* multiple-comparisons testing was used. **P* < 0.05, ***P* < 0. 01, ****P* < 0.001; ns, not significant.

**Figure S12** Phosphorylation of PFKP is required to rescue tumor growth suppressed by PLOD1 deficiency *in vivo*. (A–C) SCC-1 cells with or without PLOD1 knockout were reconstituted with PFKP-WT or PFKP-mutant and subcutaneously implanted into nude mice. Tumor progression was monitored by measuring tumor volume at the indicated time points, and tumor weight was recorded at the experimental endpoint (*n*=6). Data are presented as mean ± SD unless otherwise indicated. For comparisons among multiple groups, one-way ANOVA with *post hoc* multiple-comparisons testing was used. ****P* < 0.001; ns, not significant.

**Figure S13** Cell-surface GLUT1 analysis in response to PLOD1 modulation. (A, B) Cell-surface biotinylation followed by immunoblotting of GLUT1 in SCC-1 and SCC-23 cells expressing increasing amounts of PLOD1, together with the corresponding total lysates (*n*=3). (C, D) Cell-surface biotinylation and immunoblot analysis of GLUT1 in SCC-1 and SCC-23 cells after PLOD1 knockdown, together with the corresponding total lysates (*n*=3).

 **Figure S14** Acetate supplementation partially restores H3K27ac and associated transcriptional and phenotypic programs in PLOD1-deficient HNSCC cells. (A) Immunoblot analysis of H3K27ac in SCC-1 and SCC-23 cells following PLOD1 deletion with or without sodium acetate (1 mmol/L) supplementation (*n*=3). (B) ChIP-qPCR analysis of H3K27ac enrichment at the *TGFBR2* promoter under the indicated conditions (*n*=4). (C) qRT-PCR analysis of TGFBR2 expression in PLOD1-deficient SCC-1 and SCC-23 cells treated with PBS or sodium acetate (*n*=4). (D) Immunoblot analysis of EMT- and stemness-related markers in SCC-1 and SCC-23 cells with PLOD1 depletion, with or without sodium acetate treatment (*n*=3). (E, F) Representative spheroid formation assays and quantification of spheroid areas in cisplatin-treated SCC-1 and SCC-23 cells lacking PLOD1, with or without acetate supplementation (*n*=4). (G, H) Representative clonogenic assays and quantification of colony formation under cisplatin treatment in PLOD1-deficient SCC-1 and SCC-23 cells treated with PBS or sodium acetate (*n*=4). Data are presented as mean ± SD unless otherwise indicated. For comparisons among multiple groups, one-way ANOVA with *post hoc* multiple-comparisons testing was used. ***P* < 0. 01, ****P* < 0.001; ns, not significant.

**Figure S15** ACLY depletion reverses PLOD1-driven increases in acetyl-CoA, chromatin acetylation, and associated transcriptional and phenotypic programs. (A) Measurement of intracellular acetyl-CoA levels in SCC-1 and SCC-23 cells expressing PLOD1 with or without ACLY knockdown (*n*=4). (B) Immunoblot analysis of ACLY and H3K27ac following PLOD1 overexpression and ACLY silencing (*n*=3). (C, D) ChIP-qPCR quantification of H3K27ac enrichment at the *TGFBR2* promoter under the indicated conditions (*n*=4). (E) qRT-PCR analysis of *TGFBR2* expression in PLOD1-overexpressing cells with or without ACLY knockdown (*n*=4). (F) Immunoblot analysis of EMT- and stemness-related proteins following PLOD1 overexpression with or without ACLY knockdown in SCC-1 and SCC-23 cells (*n*=3). (G, H) Representative spheroid formation assays and quantification of spheroid areas in cisplatin-treated SCC-1 and SCC-23 cells overexpressing PLOD1 with or without ACLY depletion (*n*=4). (I, J) Representative clonogenic assays and quantification of colony formation under cisplatin treatment following PLOD1 overexpression and ACLY knockdown (*n*=4). Data are presented as mean ± SD unless otherwise indicated. For comparisons among multiple groups, one-way ANOVA with *post hoc* multiple-comparisons testing was used. ***P* < 0. 01, ****P* < 0.001; ns, not significant.

**Figure S16** PLOD1 depletion restores cisplatin sensitivity in a cisplatin-resistant PDX model. (A) Images of tumors collected from the cisplatin-resistant PDX model following treatment with LNP-siCTRL, LNP-siCTRL plus cisplatin, or LNP-siPLOD1 plus cisplatin. (B, C) Tumor weight and tumor volume measured at endpoint for the indicated treatment groups (*n*=6). Data are presented as mean ± SD unless otherwise indicated. For comparisons among multiple groups, one-way ANOVA with *post hoc* multiple-comparisons testing was used. ****P* < 0.001; ns, not significant.

**Table S1** Sequences of primers and oligos used in this study.

| Target/oligonucleotide | Sequence (5′–3′) |
| --- | --- |
| *PLOD1* | F: AAGCCGGAGGACAACCTTTTA |
|  | R: GCGAAGAGAATGACCAGATCC |
| *SOX2* | F: TACCTCTTCCTCCCACTCCA |
|  | R: ACTCTCCTCTTTTGCACCCC |
| *Nanog* | F: GCTGGTTGCCTCATGTTATTATGC |
|  | R: CCATGGAGGAAGGAAGAGGAGAGA |
| *CD133* | F: AAGCATTGGCATCTTCTATGG |
|  | R: AAGCACAGAGGGTCATTGAGA |
| *CD44* | F: CTGCCGCTTTGCAGGTGTA |
|  | R: CATTGTGGGCAAGGTGCTATT |
| *TGFBR1* | F: TCCAACTACTGGTTTACCATTGC |
|  | R: CCGCCACTTTCCTCTCCAAA |
| *TGFBR2* | F: GGTTCCTGTGTGCCCTTATT |
|  | R: TGCAACCCATGAAGGTAAAA |
| *TGFBR3* | F: GTGTTCCCTCCAAAGTGCAAC |
|  | R: AGCTCGATGATGTGTACTTCCT |
| *MMP-2* | F: AGCGAGTGGATGCCGCCTTTAA |
|  | R: CATTCCAGGCATCTGCGATGAG |
| *SNAI2* | F: TGCGATGCCCAGTCTAGAAA |
|  | R: GTGTCCTTGAAGCAACCAGG |
| *PFKP* | F: GCATGGGTATCTACGTGGGG |
|  | R: CTCTGCGATGTTTGAGCCTC |
| *ACTB* | F: ATCAAGATCATTGCTCCTCCTGAG |
|  | R: CTGCTTGCTGATCCACATCTG |
| sgPLOD1 #1 | CTTGGCGTCGCCCTTCGCTT |
| sgPLOD1 #2 | AAGGGCGACGCCAAGCCGGA |
| siACLY #1 | CGTGAGAGCAATTCGAGATTA |
| siACLY #2 | GCCTAAGTACTCTTGCCAGTT |
| shPFKP | CGAGAGGAGATTTCAAGATGC |
